# Clues of *in vivo* nuclear gene regulation by mitochondrial short non-coding RNAs

**DOI:** 10.1038/s41598-020-65084-z

**Published:** 2020-05-19

**Authors:** Marco Passamonti, Marco Calderone, Manuel Delpero, Federico Plazzi

**Affiliations:** 1 Department of Biological, Geological and Environmental Sciences, University of Bologna, Bologna, Italy; 2 Present Address: Department of Crop and Animal Sciences, Humboldt-Universität zu Berlin, Berlin, Germany

**Keywords:** Data mining, RNAi, Mitochondria, DNA sequencing, Histone analysis

## Abstract

Gene expression involves multiple processes, from transcription to translation to the mature, functional peptide, and it is regulated at multiple levels. Small RNA molecules are known to bind RNA messengers affecting their fate in the cytoplasm (a process generically termed ‘RNA interference’). Such small regulatory RNAs are well-known to be originated from the nuclear genome, while the role of mitochondrial genome in RNA interference was largely overlooked. However, evidence is growing that mitochondrial DNA does provide the cell a source of interfering RNAs. Small mitochondrial highly transcribed RNAs (smithRNAs) have been proposed to be transcribed from the mitochondrion and predicted to regulate nuclear genes. Here, for the first time, we show *in vivo* clues of the activity of two smithRNAs in the Manila clam, *Ruditapes philippinarum*. Moreover, we show that smithRNAs are present and can be annotated in representatives of the three main bilaterian lineages; in some cases, they were already described and assigned to a small RNA category (e.g., piRNAs) given their biogenesis, while in other cases their biogenesis remains unclear. If mitochondria may affect nuclear gene expression through RNA interference, this opens a plethora of new possibilities for them to interact with the nucleus and makes metazoan mitochondrial DNA a much more complex genome than previously thought.

## Introduction

Expression of nuclear genes may be enhanced or reduced by several cellular factors^1,2^, including a wide array of short non-coding RNA transcripts (sncRNAs)^3–6^. MicroRNAs (miRNAs) are possibly the most studied ones^4,7^, as they are the commonest regulators of gene expression at the posttranscriptional level^8–11^. However, other types of sncRNAs have been characterized^3–6^. For instance, piRNAs are sncRNAs that bind to PIWI Argonaute proteins, and are often associated to the maintenance of germline genome stability, transposition suppression, and chromatine remodeling^3,6,12–14^.

Many ways are known for sncRNAs to originate from transcribing DNA and to maturate towards the working molecule which will take part in the regulating complex^3,5,6,10,11,15^. The biogenesis of these small molecules normally involves a maturation from longer transcripts, as is the case for mature miRNAs, resulting from pre-miRNAs^6,9,11,16–25^, which, in turn, are obtained from pri-miRNAs^6,10,11,15,16,26–31^.

The interaction between the mature miRNA and the target mRNA takes place in the RNA induced silencing complex (RISC)^8–11,15^ and it typically involves the ‘seed’ region, i.e. bases 2–8 in the 5′ end of a miRNA^6,15,16,32^, even if other modes of interaction have been published^15^. The ‘seed’ (i.e., miRNA nucleotides 2–7) normally basepairs with a region within the 3′ UTR of the target mRNA, and many different types of seed-target interaction are known^16,32^.

The nuclear DNA is not the only DNA in the eukaryotic cell. More specifically, in animal cells, mitochondria do contain their own genome, a small, normally circular molecule encoding for a dozen of protein-coding genes, two ribosomal subunits and many tRNAs^33–35^. Mitochondrial DNA (mtDNA) is known to transcribe for sncRNAs as well. In the human mtDNA, a total of 31 sncRNAs mapping to 17 loci (prevalently tRNAs) were originally annotated^36^. These loci prevalently code for tRNAs, and it is known that tRNAs may be processed by Dicer and other RNAses in the cytoplasm into a wide array of small RNA molecules that enter RNA-silencing pathways^37,38^. Mitochondrial genome-encoded small RNAs (mitosRNAs^39^) are now known from different species^39–41^; however, although involved in many physiological processes, like anoxic response^41^ or gametogenesis^40^, mitosRNAs were never directly associated to nuclear target mRNAs. Conversely, the possible involvement of mitochondrial non-coding RNAs in nuclear regulation is an expanding research field^42,43^.

A new class of small mitochondrial highly-transcribed RNAs (smithRNAs) was recently described in the Manila clam *Ruditapes philippinarum* (Bivalvia: Veneridae) and associated to specific nuclear targets^44^. *R. philippinarum* shows a peculiar way of mitochondrial inheritance, called Doubly Uniparental Inheritance (DUI)^33,45–47^, which leads to the presence of two separate, sex-linked mitochondrial lineages, along with their highly divergent genomes (M-type for male, and F-type for female mitochondria). Putative smithRNAs were found in small RNA libraries of *R. philippinarum* gonads, mapping to both M- and F-type mitochondrial genomes, and they were analyzed *in silico*^44^. Although we used a very conservative pipeline for their validation, smithRNA functionality was never demonstrated *in vivo*.

Finally, many observations point to the fact that smithRNAs cannot be transcribed from mitochondrial pseudogenes (NUMTs): in the Manila clam, for instance, male-encoded smithRNAs are not expressed in females, which in fact lack male mitochondria, a pattern of expression that is expected for mitochondrially-encoded genes of DUI species^44^. Moreover, additional clues on the issue were recently provided^48^, further supporting the origin of smithRNAs from mitochondrial DNA.

In the present paper, we present several clues about the actual *in vivo* activity of smithRNAs in regulating nuclear gene expression in the Manila clam: loci associated to smithRNAs are indeed more conserved than unassigned regions of the mitochondrial genome, and *in vivo* interference effects were observed. Finally, we present preliminary evidence about the existence of smithRNA in distantly related eukaryotic lineages, irrespective of the biogenetic pathway, which appears to be variable or unclear.

## Results

### Conservation of smithRNA-encoding loci

When smithRNAs were firstly described in the Manila clam *Ruditapes philippinarum*^44^, nothing was known about their biogenesis and way of regulating target genes. As a preliminary hypothesis, a miRNA-like biogenesis was assumed, so that it is possible that functional sncRNAs arising from different pathways were disregarded. Conversely, stringent thresholds were used for all computations, aiming to a robust description of at least a subset of existing smithRNAs. Nonetheless, at that point, bioinformatics was the only source of data for characterization and actual functionality of these smithRNAs.

In the present paper, we focused on gathering clues of functionality of these sncRNAs in regulating nuclear genes. Specifically, we devised three different lines of experimental evidence to support their functionality: (i) by sequence conservation, (ii) by *in vivo* observation of interference effects, and (iii) by distribution among metazoans.

A first rationale was as follows: many (putative) smithRNAs map to mitochondrial unassigned regions (URs)^44^, that are typically abundant in bivalve mtDNAs^49,50^. URs should experience relaxed selective pressure with respect to surrounding genomic regions, unless they are actually functional in some way: for instance, unless they do encode for a sncRNA.

**Figure 1:**
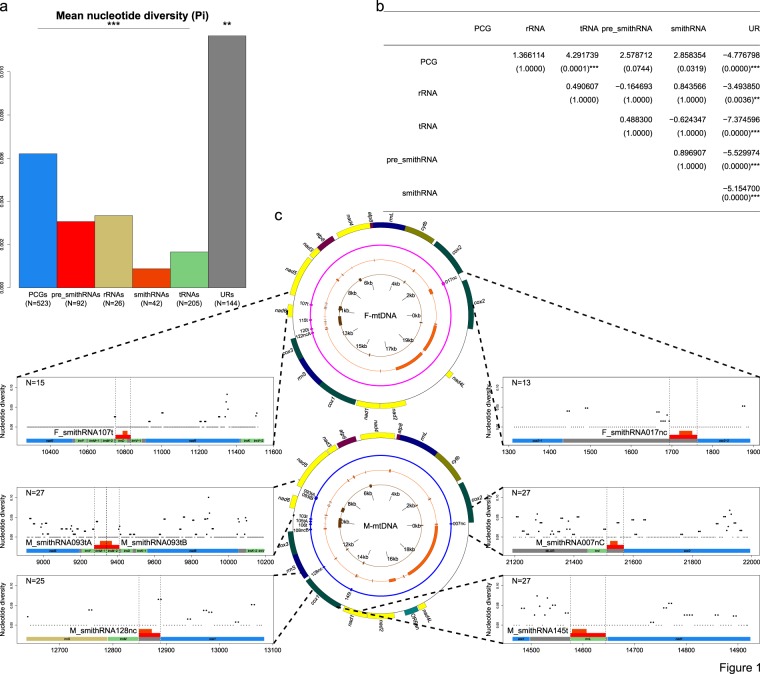
Conservation of Manila clam smithRNA loci. (**a**), mean nucleotide diversity (π) for five type of coding regions, including putative pre_smithRNAs and smithRNAs, and URs. (**b**), results of Dunn’s test for the six types of regions; sample sizes are shown in (**a**). (**c**), nucleotide diversity over a sliding window in six genomic regions: colours as in a. Windows with nucleotide diversity equal to 0 are shown with small circles, while those with nucleotide diversity greater than 0 are shown with larger circles. Annotation of mtDNA is reported: from inner to outer, circles bear tRNAs, URs, smithRNAs, and PCGs.

To test the hypothesis of functionality of smithRNAs, we sequenced smithRNA-encoding loci and flanking regions in 15 female and 27 male specimens of *R. philippinarum* and partitioned sequenced regions into different functional regions: protein-coding genes (PCGs), rRNA-encoding genes (rRNAs), tRNA-encoding genes (tRNAs), regions predicted to transcribe for a pre-smithRNA, regions predicted to transcribe for a mature smithRNA, and (re-annotated) URs. Nucleotide diversity (π) was computed along a sliding window on the resulting alignments (Fig. 1).

Levels of nucleotide diversity of the five types of coding regions were not significantly different; conversely, they were all significantly different from the level of nucleotide diversity measured within URs (Kruskal-Wallis rank sum test with 5 degrees of freedom, χ^2^ = 67.8566, P = 0; two-tailed pairwise Dunn’s test with Bonferroni correction; Fig. 1). The only exception to this was the comparison between PCG and tRNA regions: however, the relatively high level of diversity detected within PCGs is largely due to synonymous mutations, which experience relaxed selective pressure (Supplementary Table S1).

In sum, the conservation of putative smithRNA loci is comparable to that of other well-known functional mitochondrial loci, and, even if some of them were originally annotated within URs, nucleotide diversity significantly drops for smithRNA-coding regions with respect to the remainder of URs (Fig. 1).

### *In vivo* assay of M_smithRNA106t and 145t

The original *in silico* predictions allowed us to identify one or more nuclear targets for all the 14 putative smithRNAs^44^. The only putative target of M_smithRNA106t, which is annotated between *cox3* and *atp6* on the male mtDNA (Fig. 1), is the clam homolog of a human Histone-lysine N-methyltransferase (SETD2; Uniprot entry Q9BYW2). Therefore, when M_smithRNA106t is present, cells are expected to show reduced levels of methylation on histone H3^51^.

100 μg of M_smithRNA106t were injected into 61 clams at the concentration of 100 ng/μL; the same volume (1 mL) of ddH_2_O was injected into 60 control specimens. Levels of methylation were assayed 2 h and 24 h after injection, along with levels of methylation in 34 untreated specimens.

**Figure 2:**
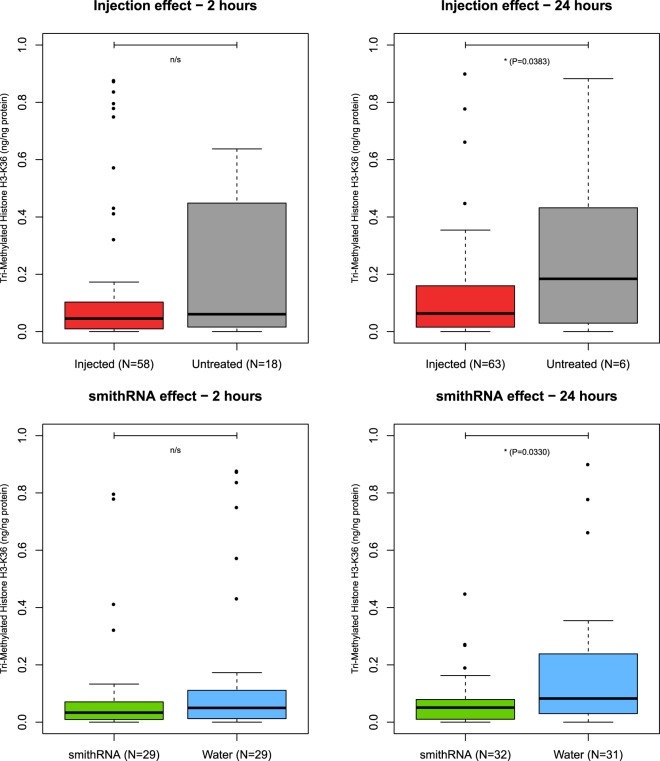
Effect of the injection of M_smithRNA106t on Manila clams. Injected, individuals injected with either M_smithRNA106t or ddH_2_O; untreated, uninjected individuals. The black line is the median; the two hinges of the box approximate the first and the third quartile; whiskers extend to a roughly 95% confidence interval.

The injection itself entailed a significant reduction of methylation levels after 24 h (one-tailed Mann-Whitney test, W = 358.5, P = 0.0383); however, methylation levels are significantly lower in individuals injected with the smithRNA targeting a methyltransferase, when compared to control individuals injected with distilled water (one-tailed Mann-Whitney test, W = 362, P = 0.033; Fig. 2). The smithRNA-triggered effect seems especially evident in female specimens (Supplementary Fig. S1). Recall that it is not possible to *a priori* predict the sex of specimen, groups, including the untreated group, show some differences in size; however, the use of nonparametric tests accounts for the different dimension of resulting groups.

M_smithRNA145t was associated with three different targets^44^; among them, a homolog of another human Histone-lysine N-methyltransferase (SETD8; Uniprot entry Q9NQR1), and the homolog of the human DNA polymerase epsilon subunit 3 (POLE3/CHRAC17; Uniprot entry Q9NRF9), which has been involved into the process of chromatin remodeling, and particularly to histone acetylation^52^. Again, when M_smithRNA145t is present, cells are thus expected to show altered levels of histone acetylation.

As above, 100 μg of M_smithRNA145t were injected into 50 clams, while 41 control clams were injected with F_smithRNA107t (which is not expected to affect chromatin remodelling^44^) and 53 clams were not treated. We decided to use a different ssRNA as control in this case to rule out the injection effect which was observed in the previous experiment: both groups were injected with a ssRNA, therefore differences in acetylation levels ought to be associated to the specific ssRNA itself.

**Figure 3:**
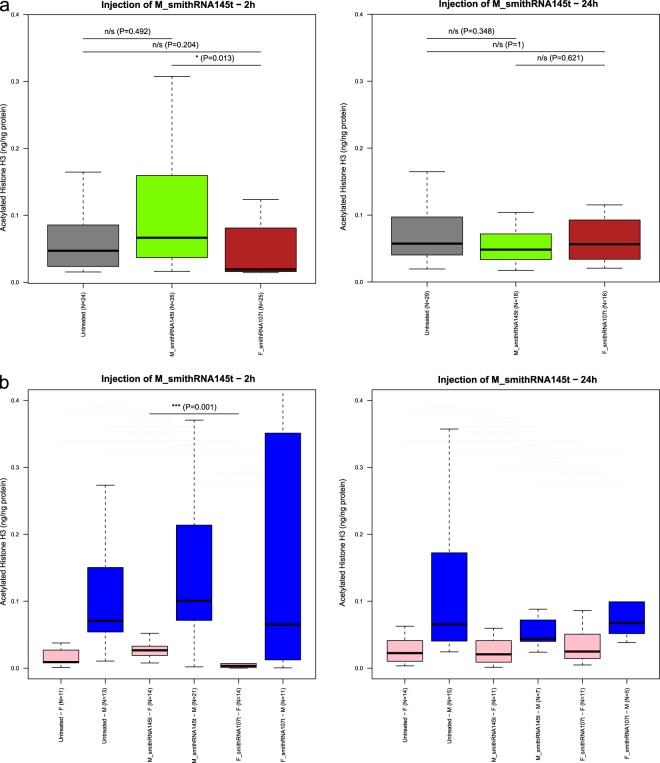
Effect of the injection of F_smithRNA107t and M_smithRNA145t on Manila clams. Untreated, uninjected individuals. (**a**), two sexes together; (**b**), injection effect shown by sex. Only significant pairwise Dunn’s test comparisons are shown for the sake of clarity; for complete results, see Supplementary Table S2. The black line is the median; the two hinges of the box approximate the first and the third quartile; whiskers extend to a roughly 95% confidence interval.

In this case, we observed a significant increase of histone H3 acetylation 2 hours after injection in specimens injected with M_smithRNA145t with respect to control and untreated specimens (Kruskal-Wallis rank sum test with 2 degrees of freedom, χ^2^ = 6.8685, P = 0.03; two-tailed pairwise Dunn’s test with Bonferroni correction); however, this effect became negligible after 24 h (Fig. 3). Again, when considering females and males separately, the significance holds for female specimens only (Kruskal-Wallis rank sum test with 2 degrees of freedom, χ^2^ = 11.3299, P = 0; two-tailed pairwise Dunn’s test with Bonferroni correction; Supplementary Table S2). The high complexity of the involvement of POLE3/CHRAC17 in chromatin remodeling may well lead to the emergence of the observed upregulation effect by means of M_smithRNA145t, instead of the expected silencing.

### Distribution of smithRNAs among animals

Wide-scale characterization of smithRNAs in different metazoan clades are beyond the aim of the present paper, which mainly aims to demonstrate the *in vivo* functionality of these sncRNAs. However, smithRNAs are not ‘weird RNA species’ of (at most) some clam species. We applied the same pipeline of the original study^44^ to other metazoan species in order to conservatively recover good smithRNA candidates from other systems. As smithRNAs were originally described from gonad tissues (given the peculiarities of the DUI phenomenon), putative smithRNAs were also identified from gonad samples.

**Figure 4:**
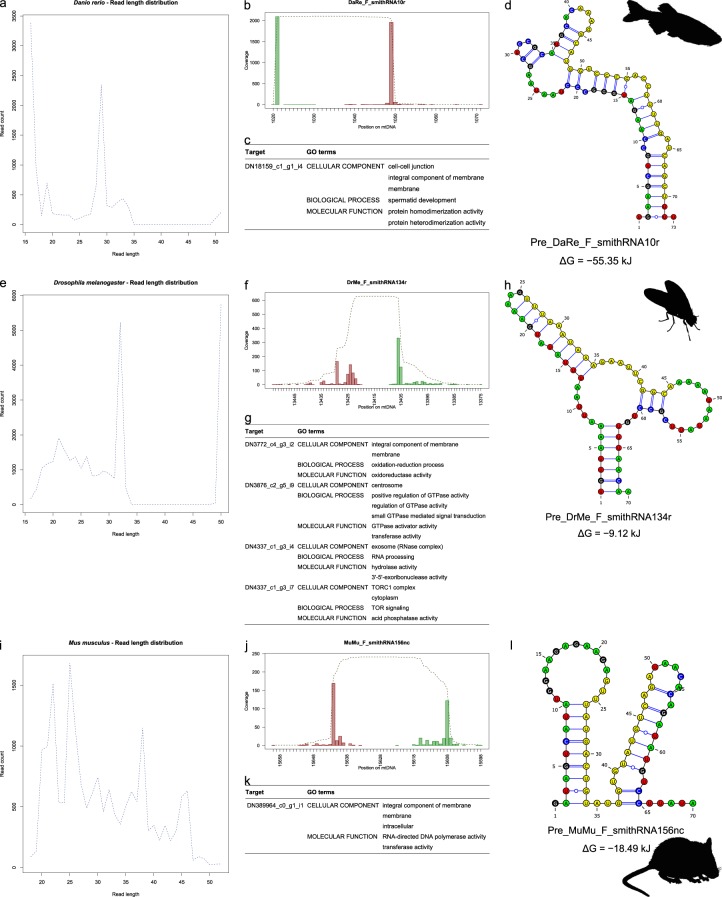
Three putative smithRNAs from *Danio rerio* (**a–d**), *Drosophila melanogaster* (**e–h**), and *Mus musculus* (**i–l**). (**a,e,i**): read length distribution for clusters with size greater than 200, whose centroid were retained for downstream analyses; (**b,f,j**): total and 5′/3′ coverage on the mitochondrial genome. 5′ coverage is shown in green and 3′ coverage is shown in red; note that two putative smithRNAs would encode on the minus strand, hence the inversion of the horizontal axis (**f,j**). (**c,g,k**): top scoring GO terms associated with predicted targets; for complete ARGOT results, see Supplementary Table S3. (**d,h,l**): secondary structure of the genomic contexts. Mature putative smithRNAs are shown in yellow.

The fruitfly, *Drosophila melanogaster*, belongs to Ecdysozoa, while *R. philippinarum* belongs to Lophotrochozoa. We detected 2 putative smithRNAs from *Dr. melanogaster* ovaries and 8 putative smithRNAs from *Dr. melanogaster* testes (Fig. 4a–d). The zebrafish, *Danio rerio*, and the mouse, *Mus musculus*, are widely used model species as well; contrastingly with the two aforementioned protostome species, they belong to Deuterostomia. We detected 3 putative smithRNAs from *Da. rerio* ovaries and 3 putative smithRNAs from *Da. rerio* testes (Fig. 4e–h). Contrastingly, only one putative smithRNA was detected in *M. musculus* ovaries; however, when relaxing the read cluster size threshold from 200 to 50, we retrieved four more putative smithRNA in mouse ovaries and one putative smithRNA in mouse testes (Fig. 4i–l).

Complete details on the annotation of putative smithRNAs in *Dr. melanogaster*, *Da. rerio*, and *M. musculus* are given in Supplementary Dataset S1, Supplementary Dataset S2, and Supplementary Table S3; here we provide an example from each model species. F_smithRNA134r from *Dr. melanogaster* is predicted to target four transcripts, involved in signal transduction and RNA processing. F_smithRNA10r from *Da. rerio* has a single predicted target, connected to cell junction. F_smithRNA156nc from *M. musculus* has a single predicted target as well, possibly an integral component of membrane.

Putative smithRNAs from *R. philippinarum* and these three model species were queried against three different sncRNA databases: miRbase^53–58^, piRBase^59–62^, and fRNAdb^63,64^. Interestingly, all the smithRNAs identified in the mouse have been described as piRNAs elsewhere, and four out of six do map on the mouse mitochondrial genome (Supplementary Table S4). Conversely, only five out of ten fly smithRNAs were found in piRBase, again mapping on the mitochondrial genome. All *Drosophila* smithRNAs have similarities with fly small RNAs in fRNAdb, though sometimes with high e-values. Finally, no significant similarities were detected for zebrafish and Manila clam smithRNAs in these databases, with the exception of low-similarity hits for three out of six zebrafish smithRNAs that were associated to ape/plant miRNAs and a mouse mitochondrial piRNA, in all cases with large e-values (Supplementary Table S4).

We carried out the same analysis of nucleotide diversity that was carried out for *R. philippinarum* on available complete mitochondrial genomes of *Dr. melanogaster* and *M. musculus* (Supplementary Table S5). Again, the amount of nucleotide diversity in unassigned regions is significantly higher than in any other coding region of the genome, including regions where putative smithRNAs and pre_smithRNAs were mapped; moreover, with minor exceptions, the amount of nucleotide diversity is comparable among all coding regions (Kruskal-Wallis rank sum test with 5 degrees of freedom, χ^2^ = 303.0833 and 192.0627 for fruit fly and mouse, respectively, P = 0; two-tailed pairwise Dunn’s test with Bonferroni correction; Supplementary Fig. S2).

## Discussion

Our analysis evidenced that smithRNA loci on both mitochondrial genomes of *Ruditapes philippinarum* show variability levels similar to coding loci. The conservation to an extent comparable with other coding regions is an evidence of selective constraints acting on these regions, which in turn constitutes a strong clue of functionality for these loci.

Furthermore, both injection experiments provided the first *in vivo* evidence of regulation of nuclear genes through small RNAs encoded by the mitochondrion; moreover, since the targets are broad-range epigenetic effectors, these smithRNAs may have a major impact on nuclear genome regulation. Finally, we showed that smithRNAs are present and can be annotated in representatives of the three main bilaterian lineages.

The characterization of molecular details of sncRNA biogenesis and silencing mechanism, as well as their scope on different organisms, is a growing research field. There is virtually no physiological process that has not been somehow linked to these molecules, including tumorigenesis, immune and stress response, reproduction, and development^3,4,6,65–69^.

Besides clams^44^, small RNAs coded by the mitochondrion (mitosRNAs) were recently described in plants^70^ and many metazoans, including humans^36,39^, mice^39,40^, chickens^71^, sharks, teleost fishes, turtles, and frogs^41^. Different roles have been proposed for these mitochondrially-encoded RNAs, ranging from anoxia tolerance^41^ to gametogenesis and fertilization^40^; however, to our knowledge, the only targets that were explicitly linked to mitochondrial sncRNAs are mitochondrial PCGs themselves^36,39,71^. Conversely, the ability of mitochondria to shape nuclear gene expression has already been noted^72,73^. Thus, smithRNAs may be the tile to complete this puzzle about retrograde (i.e., mitochondrion-to-nucleus) signalling^74–76^.

Mitochondrially-encoded smithRNAs must exit the mitochondrion to regulate nuclear transcripts in the cytoplasm, if not enter the nucleus itself. In the present paper we do not provide direct evidence of the presence of smithRNA molecules outside the mitochondrion; however, mitochondrial RNA outside the source organelle has been already observed. Several mitochondrially-encoded tRNAs were found in the cytoplasm of human cells^77^, in one case associated to Ago2, the protein involved in the final cleavage of many sncRNA biogenetic pathways. A release of mitochondrial material has been observed in *R. philippinarum*^78^, and this would provide a very obvious mechanism for smithRNAs to enter cytoplasm. Long non-coding RNAs transcribed by the mitochondrial genome were reported in the nucleus^79–81^, and therefore the presence of short non-coding ones would not be surprising.

Here we provide the first experimental data on smithRNA functionality on specific nuclear targets, demonstrating that in eukaryotic cells small mitochondrial transcripts do exit the mitochondrion to exert regulatory roles. Moreover, targets that are investigated here have a potential genome-scale effect, being epigenetic regulators: thus, not only can mitochondria affect nuclear expression, but also their scope is possibly massive.

Some putative smithRNAs were already described as piRNAs (all the putative smithRNAs we detected in *Mus musculus* and half of the putative smithRNAs we detected in *Drosophila melanogaster*). Previous reports showed that most mitosRNAs are indeed piRNAs in *M. musculus*^40^, and piRNAs are a class of sncRNAs which is typically associated to germline cells^13,82,83^, a fact which was recently confirmed in mollusks^84^. Moreover, the PIWI biogenetic pathway, involved in piRNA biogenesis^85,86^ is associated to mitochondria^87^ and piRNAs were already connected to mitochondrial communications to the nucleus^79^. Consistently with our mapping, piRNAs were previously found to originate from mitochondrial tRNAs and rRNAs^83^.

However, many other putative smithRNAs have unclear affinities with already described sncRNAs (half of the putative smithRNAs we detected in *Dr. melanogaster*) or no affinity at all (*Danio rerio*; *R. philippinarum*; Supplementary Table S4). It is tempting to conclude that smithRNAs transcribed by the mitochondrial genome were exapted from many different biogenetic pathways, most notably from that typical of piRNAs. Similarly, they may act with different mechanisms, either silencing or enhancing nuclear genes, as was observed in our *in vivo* assays.

These recently discovered tools may thus be heterogeneous in terms of biogenesis and activity, yet they share a functional role: a retrograde signaling directed towards nuclear genes. Moreover, given the available, stringent bioinformatic pipeline, they normally appear to be highly expressed. In fact, mitochondrial genomes already have some features (including tRNAs and secondary structures in intergenic regions) that are likely to exapt towards the evolution of regulatory RNAs. Put in other words, we regard to smithRNAs as a class of mitosRNAs which by definition affect nuclear gene expression to some extent.

These small genetic elements provide an effective way for mitochondria to largely influence nuclear functioning: while more smithRNAs should be experimentally validated in the future, it is now important to achieve a clearer picture of the distribution and abundance of these RNAs among living beings. Our present findings are necessary conservative, in that miRNA-like biogenesis and action mechanism were assumed, and it is likely that many smithRNAs were overlooked. Yet, our results highlight the presence of smithRNAs in the three major lineages of bilaterians (Ecdysozoa, Lophotrochozoa, and Deuterostomia), suggesting that the emergence of these regulatory elements is (at least) as ancient as bilaterians and that they represent a widespread mechanism for mitochondria to influence nuclear expression.

## Methods

### Conservation of smithRNA-encoding loci

DNA from gametes of 15 female and 27 male *Ruditapes philippinarum* clams was collected and extracted as in^50^. PCR amplifications of mitochondrial regions flanking putative smithRNAs were carried out on a 2720 Thermal Cycler (Applied Biosystem) with GoTaq Flexi DNA Polymerase (Promega), as follows: 10 μL 5 × Green GoTaq Flexi Buffer, MgCl_2_ (3 mM), nucleotides (800 μM each), primers (500 nM each), 1 U GoTaq DNA Polymerase, 40 ng template DNA, ddH_2_O up to 50 μL. Primers were designed using Primer3^88–90^ and are listed in Supplementary Table S6. PCR cycles were set following manufacturer’s instructions, as follows: initial denaturation at 95 °C for 2′; 35 cycles of denaturation at 95 °C for 1′, annealing at 48–56 °C for 1′, and extension at 72 °C for 1′; final extension at 72 °C for 5′. PCR results were visualized using a 1% electrophoresis agarose gel stained with ethidium bromide. Amplicons were purified through a standard isopropanol protocol and sequenced at the Macrogen Europe facilities. Electropherograms were edited using MEGA7^91^. Mean nucleotide diversity is defined as

$$\hat{\pi }=\frac{N}{N-1}\mathop{\sum }\limits_{i=1}^{N}\,\mathop{\sum }\limits_{j > i}^{N}\,{p}_{i}{p}_{j}{\pi }_{ij}$$

where *N* is the number of sequences, *p*_*i*_ is the frequency of the *i*-th haplotype, *p*_*j*_ is the frequency of the *j*-th haplotype and *π*_*ij*_ is the uncorrected (p) distance between haplotypes *i* and *j*. Mean nucleotide diversity was computed over a 10-bp sliding window with 5-bp steps using the software Variscan^92^; general data organization, the Kruskal-Wallis rank sum test, and the two-tailed pairwise Dunn’s test were carried out using the software R^93^ and the package dunn.test^94^. Mitochondrial DNAs were drawn using the software GenomeVx^95^.

### *In vivo* assay of M_smithRNA106t and 145t

*R. philippinarum* specimens were sampled in Italy (Goro) in June/July 2017 and 2018 during two sampling campaigns. Individuals were collected during the reproductive season. After sampling, clams were placed in different beakers containing reverse osmosis water with Red Sea Coral Pro aquariology sea salt (Red Sea Europe).

1,500 nmol of custom ssRNAs corresponding to M_smithRNA106t, M_smithRNA145t, and F_smithRNA107 were synthetized at the Integrated DNA Technologies, Inc. facilities. ssRNAs were resuspended in ddH_2_O to a final concentration of 100 ng/μL. 1 mL of ssRNA or ddH_2_O was injected into clams by slightly widening valves’ opening and inserting the sterile 2.5-mL syringe needle into the mature gonad, immediately above the mollusk’s foot. Two or twenty-four hours after the injection, specimens were collected and sexed by microscopic dissection of gonadal tissue. Gonadal tissues were then sampled and stored at −80 °C for histone extraction.

Total histone extraction and tri-methylation quantification were carried out using the EpiQuik Total Histone Extraction Kit and the EpiQuik Global Tri-Methyl Histone H3-K36 Quantification Kit (Colorimetric) (EpiGentek); acetylation quantification was carried out using the EpiQuik Global Histone H3 Acetylation Assay Kit (EpiGentek), following manufacturer’s instruction. Protein concentration was quantified using the Quick Start Bradford Protein Assay (BioRad) on a NanoGenius Photometer Onda. Colorimetric assays were quantified on a Benchmark Microplate Reader (Bio-Rad). General data organization, the Mann-Whitney test, the Kruskal-Wallis rank sum test, and the two-tailed pairwise Dunn’s test were carried out using the software R and the package dunn.test.

### Distribution of smithRNAs among animals

The pipeline of Pozzi and colleagues^44^ was strictly followed in order to obtain comparable results from *Danio rerio*, *Drosophila melanogaster*, and *Mus musculus*. An overview of the bioinformatic pipeline is provided as Supplementary Fig. S3.

Raw reads were downloaded from the GenBank repository: SRA Accession Numbers are listed in Supplementary Table S7. Reads were filtered with Trimmomatic 0.38^96^ and potential contaminations were discarded using kraken2 2.0.8^97^. Settings of the filtering stage were ILLUMINACLIP:2:30:10 AVGQUAL:20 LEADING:3 TRAILING:3 SLIDINGWINDOW:4:20 for smallRNA-Seq and ILLUMINACLIP:2:30:10 AVGQUAL:20 LEADING:3 TRAILING:3 SLIDINGWINDOW:25:33 MINLEN:75 (if suitable) for RNA-Seq; the database used for the software kraken2 was the standard database built on the 20^th^ of June, 2019. Transcriptomes were assembled using the software Trinity 2.6.6^98^ with the–no_normalize_reads option. The completeness of transcriptomes was assessed using the online tool gVolante^99^ against the relevant database. ORFs and relative 3’ UTRs were identified using the software ExUTR 0.1.0^100^ against the Swissprot database with options -x 2500 -m 1.

Small transcriptomes’ reads were mapped against the *Da. rerio*, *Dr. melanogaster*, and *M. musculus* mitochondrial genomes (GenBank Accession Numbers NC_002333.2, KJ947872.2, and AY172335.1, respectively) using the software Bowtie2 2.3.0^101^ in the end-to-end mode. The maximum number of tolerated mismatches was set to 1 using the options -N 1 -i C,1 -L 18. Reads mapping to the mitochondrial genome were mapped against the latest release of the nuclear genome (GenBank Accession Numbers GCA_000002035.4, GCA_000001215.4, and GCA_000001635.8, respectively), again using end-to-end Bowtie2 alignments (options: -i C,1 -L 22). Reads not mapping on the nuclear genome were mapped again on the mitochondrial genome (as before) and analyzed further. Clusters of reads were created using USEARCH 11.0.667^102^ setting the identity score to 0.99 (which, for short smallRNA-Seq reads, means total identity).

According to our original publication^44^, multiple requirements were mandatory for a centroid of a cluster to be considered a putative smithRNA: (1) a cluster size greater than 200; (2) sharp 5′ and 3′ coverage measured with bedtools 2.26.0^103^, meaning that the sncRNA has strongly preferred starting and ending sites for transcription; (3) perfect matching of nucleotides 4–10 of the centroid with a 3′ UTR from the transcriptome, accounting for the phenomenon of seed shift (see^44^ for details); (4) at least 11 matches between the centroid and a 3′ UTR, taking advantage of the EMBOSS 6.6.0 suite^104^ and following alignment scores computed by BLAST + 2.6.0^105^ using options -task blastn-short -strand minus; (5) a ΔΔG score lower than −9 kJ for the centroid-target UTR interaction, as computed by the software PITA 6^106^ considering 3 and 15 nucleotides as 5′ and 3′ flanking sites, respectively; (6) a Gibbs free energy score lower than −20 kJ for the centroid-mRNA duplex, as computed by RNAhybrid 2.1.2^107^ (options: -f 3,10 -e -20 -p 0.05 -s 3utr_fly -t).

Centroids meeting all these requirements were considered *bona fide* putative smithRNAs and the secondary structure of the genomic context was computed using RNAfold from the ViennaRNA 2.4.13 package^108^, setting the folding temperature to 25 °C. Most putative smithRNAs fell into larger ribosomal genes, and the pre_smithRNA regions were arbitrarily set to the 70-bp long region centered on the putative smithRNA sequence, which gives an approximation of secondary structures in that genomic context. The only exception to this is DaRe_F_smithRNA10r: mapping at the very beginning of a ribosomal gene, we included the 3’ region of the upstream tRNA gene, in order to have the putative smithRNA approximately in the middle of the considered sequence. For those putative smithRNAs mapping to a tRNA gene, the whole tRNA region, along with flanking unassigned nucleotides, was used as pre_smithRNA. Secondary structures were drawn using the software VARNA^109^.

Putative smithRNAs were named according to our previous publication^44^: after the species abbreviation (‘DaRe’, ‘DrMe’, or ‘MuMu’), the first letter (either ‘F’ or ‘M’) denotes the sex of the individual (and, thus, the gonad), while numbers refer to the 100-based position of the smithRNA on the mtDNA and ‘r’, ‘t’, or ‘nc’ stands for ‘mapping to a ribosomal gene’, ‘mapping to a tRNA gene’, or ‘mapping to a non-coding region’, respectively. GO terms were associated to predicted targets of putative smithRNAs using ARGOT2^110–112^.

To investigate the variability of loci coding for putative smithRNAs in model species, 43 complete mitochondrial genomes of *D. melanogaster* and 162 complete mitochondrial genomes of *M. musculus* were downloaded from GenBank using CLC Main Workbench (QIAGEN), aligned by region using Muscle^113^, and concatenated. *Da. rerio* was excluded because of the paucity of available annotated complete mitochondrial genomes. As above, we used Variscan to compute nucleotide diversity along a sliding window and the R environment for general data organization, the Kruskal-Wallis rank sum test, and the two-tailed pairwise Dunn’s test.

## Supplementary information

Supplementary information.

Supplementary information2.

Supplementary information3.

Supplementary information4.

## Data Availability

The *Ruditapes philippinarum* sequences generated and analysed during the current study are available in the DDBJ/ENA/GenBank repository under the Accession Numbers: MN814873-MN815006. Putative *Drosophila melanogaster*, *Danio rerio*, and *Mus musculus* smithRNA sequences are available in the Third Party Annotation Section of the DDBJ/ENA/GenBank databases under the accession numbers TPA: BK010906-15, BK011029-34, and BK011035-40, respectively. All custom-tailored scripts used for data analysis are available from the corresponding author on reasonable request.
