# Supplementary material for: Clues of in vivo nuclear gene regulation by mitochondrial short non-coding RNAs
Source: Sci Rep. 2020 May 19;10:8219. doi: 10.1038/s41598-020-65084-z (PMC7237437; doi:10.1038/s41598-020-65084-z)
Supplement: Supplementary file 1 — Supplementary information. [file 41598_2020_65084_MOESM1_ESM.pdf]

Clues of *in vivo* nuclear gene regulation by mitochondrial short non-coding RNAs

Marco Passamonti

Marco Calderone

Manuel Delpero

Federico Plazzi

Supplementary Dataset S1. Newly annotated smithRNAs in *Danio rerio*, *Drosophila melanogaster*, and *Mus muculus*. For naming conventions, see Methods.

Supplementary Dataset S2. Secondary structure of genomic context. The secondary structure of the genomic context of each smithRNA provided in Supplementary Dataset S1 online is shown in the dot-bracket (Vienna) format; the Gibbs free energy is provided within parentheses, after the secondary structure. See Methods for naming conventions.

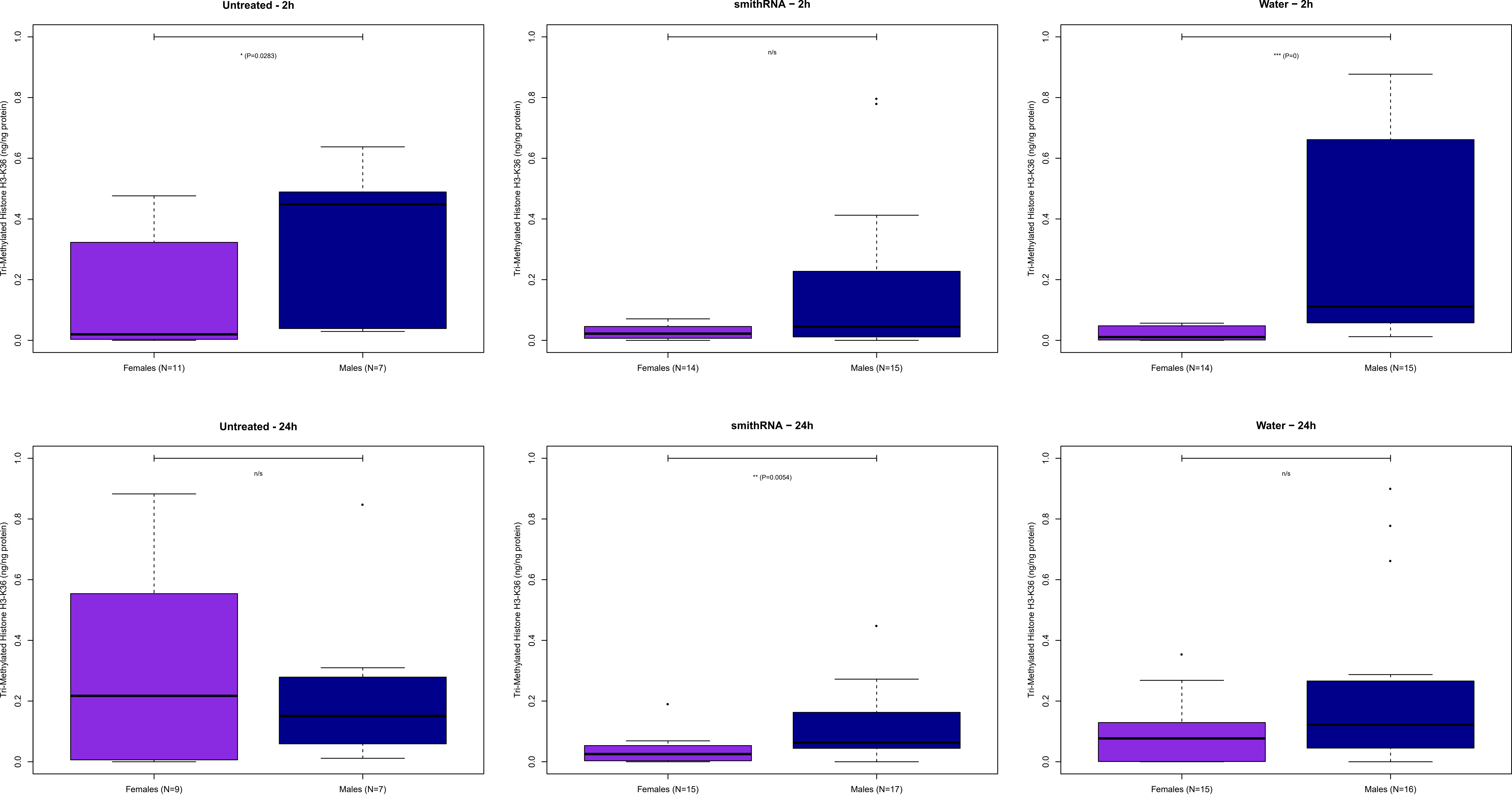

Supplementary Figure S1. Effect of the injection of M_smithRNA106t on Manila clams subdivided by time after treatment and sex. Untreated, uninjected individuals; smithRNA, individuals injected with M_smithRNA106t; water, individuals injected with ddH_2O. The black line is the median; the two hinges of the box approximate the first and the third quartile; whiskers extend to a roughly 95% confidence interval.

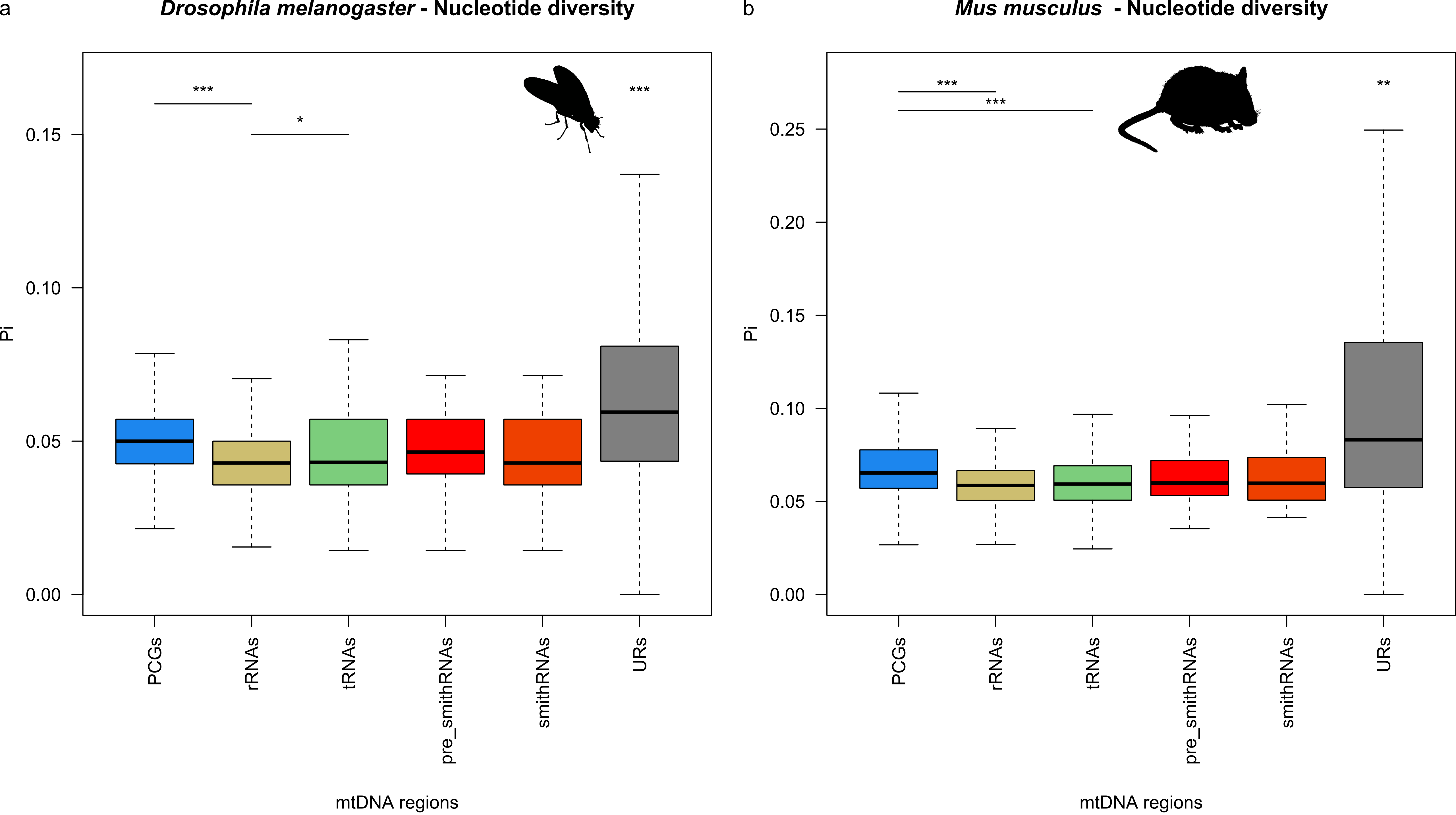

Supplementary Figure S2. Nucleotide diversity (Pi) computed along a sliding window over an alignment of (a) 43 *Drosophila melanogaster* and (b) 162 *Mus musculus* complete mitochondrial genomes. All significant two-tailed pairwise Dunn's test comparisons have an associated P<0.0133 for *Dr. melanogaster* and P<0.0026 for *M. musculus*. The black line is the median; ther two hinges of the box approximate the first and the third quartile; whiskers extend to a roughly 95% confidence interval.

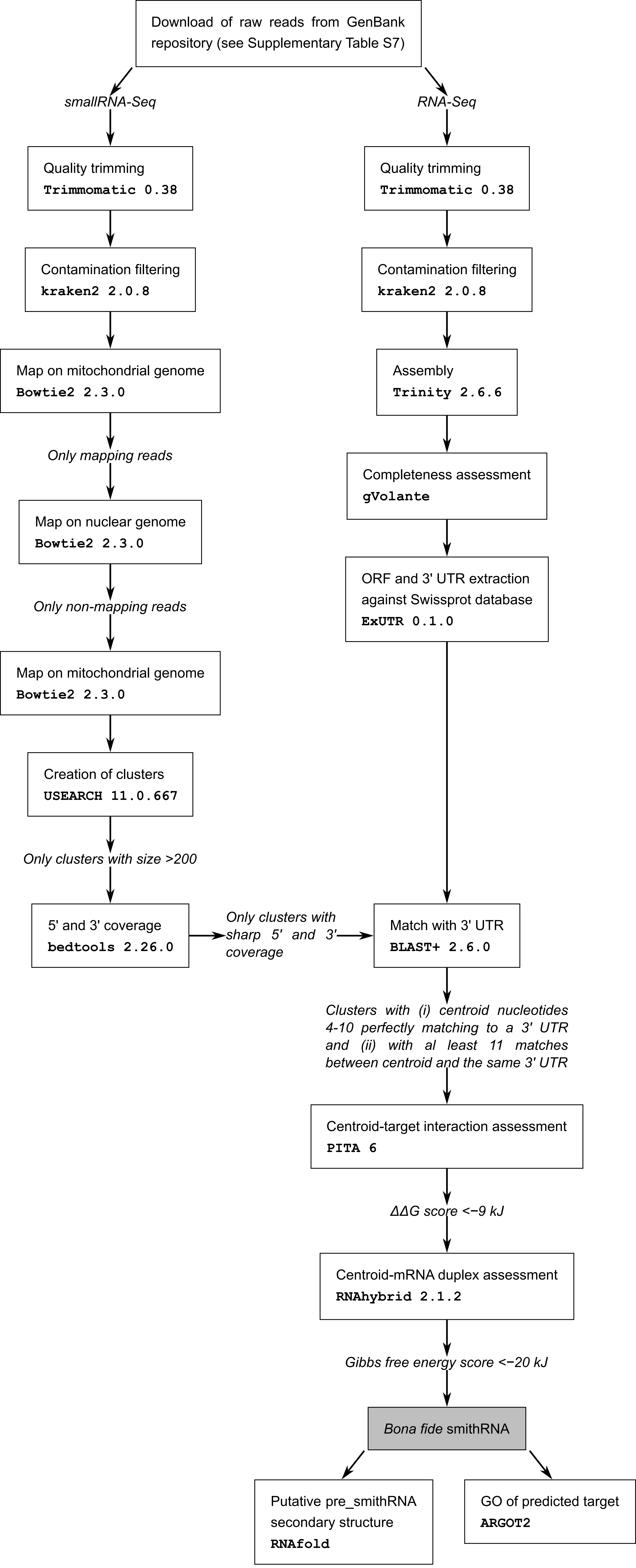

Supplementary Figure S3. The bioinformatic pipeline used for the detection of putative smithRNAs in model species.

Supplementary Table S1. Synonymous and nonsynonymous substitutions in PCGs flanking pre-smithRNA loci.

Gene	Length of amplified region	Substitutions		Total
		synonymous	nonsynonymous	
F-*nad5* (N=15)	249 bp	1	0	1
F-*nad6* (N=15)	492 bp	7	8	15
F-*cox2-2* (N=13)	132 bp	0	1	1
M-*cox1* (N=25)	229 bp	6	1	7
M-*nad1* (N=27)	281 bp	6	1	7
M-*nad5* (N=27)	286 bp	12	17	29
M-*nad6* (N=27)	492 bp	11	4	15

Supplementary Table S2. Pairwise Dunn's test levels of significance for treatment with M_smithRNA145t.

Hours after treatment	Sex	Comparison[1]	Dunn's test level of significance
2	F	Untreated–M_smithRNA145t	0.145
		Untreated–F_smithRNA107t	0.002
		M_smithRNA145t–F_smithRNA107t	0.001***
	M	Untreated–M_smithRNA145t	0.794
		Untreated–F_smithRNA107t	1.000
		M_smithRNA145t–F_smithRNA107t	0.662
24	F	Untreated–M_smithRNA145t	1.000
		Untreated–F_smithRNA107t	0.852
		M_smithRNA145t–F_smithRNA107t	0.576
	M	Untreated–M_smithRNA145t	0.657
		Untreated–F_smithRNA107t	1.000
		M_smithRNA145t–F_smithRNA107t	0.605

[1] Untreated, individuals not injected; M_smithRNA145t, individuals injected with 100 μg of M_smithRNA145t (involved in histone acetylation regulation); F_smithRNA107t, individuals injected with 100 μg of F_smithRNA107t (not involved in histone acetylation regulation).

Supplementary Table S3. GO terms associated with predicted targets of newly annotated smithRNAs provided in Supplementary Dataset S1 online.

Supplementary Table S4. Hits of putative smithRNAs detected in *Danio rerio*, *Drosophila melanogaster*, and *Mus musculus*. Sequences were queried against miRbase (http://www.mirbase.org/), piRBase (http://www.regulatoryrna.org/database/piRNA/index.html), and fRNAdb (http://togodb.biosciencedbc.jp/togodb/view/frnadb_summary#en). When a complete identity was found, the e-value is not provided.

smithRNA	Hit[1]	Organism	Locus[2]	e-value	Database
DaRe_F_smithRNA32r	bdi-miR162	*Brachypodium distachyon*	3: 49592002-49592134 [+]	1.6	miRBase
DaRe_F_smithRNA32r	pab-miR162a	*Picea abies*	MA_230619: 2413-2531 [-]	1.6	miRBase
DaRe_M_smithRNA10t	hsa-miR-1286	*Homo sapiens*	chr22: 20249134-20249211 [-]	5.9	miRBase
DaRe_M_smithRNA10t	ptr-miR-1286	*Pan troglodytes*	chr22: 6839054-6839130 [-]	5.9	miRBase
DaRe_M_smithRNA10t	ppy-miR-1286	*Pongo pygmaeus*	chr22: 16146328-16146402 [-]	5.9	miRBase
DaRe_M_smithRNA10t	ggo-miR-1286	*Gorilla gorilla*	chr22: 3520215-3520325 [-]	5.9	miRBase
DaRe_M_smithRNA49t	piR-mmu-3852	*Mus musculus*	chrM:3848-3879:+	0.098	fRNAdb
DrMe_F_smithRNA127r	FR508480	*Drosophila*		0.002	fRNAdb
DrMe_F_smithRNA127r	FR486950	*Drosophila*		0.002	fRNAdb
DrMe_F_smithRNA127r	FR476767	*Drosophila*		0.002	fRNAdb
DrMe_F_smithRNA127r	FR454233	*Drosophila*		0.002	fRNAdb
DrMe_F_smithRNA127r	FR430424	*Drosophila*		0.002	fRNAdb
DrMe_F_smithRNA127r	FR428652	*Drosophila*		0.002	fRNAdb
DrMe_F_smithRNA127r	FR426856	*Drosophila*		0.002	fRNAdb
DrMe_F_smithRNA127r	FR505462	*Drosophila*		0.006	fRNAdb
DrMe_F_smithRNA127r	FR505461	*Drosophila*		0.006	fRNAdb
DrMe_F_smithRNA127r	FR497675	*Drosophila*		0.006	fRNAdb
DrMe_F_smithRNA127r	FR476766	*Drosophila*		0.006	fRNAdb
DrMe_F_smithRNA127r	FR414623	*Drosophila*		0.006	fRNAdb
DrMe_F_smithRNA127r	FR497134	*Drosophila*		0.025	fRNAdb
DrMe_F_smithRNA134r	FR408244	*Drosophila*		4e-04	fRNAdb
DrMe_M_smithRNA128r	piR-dme-2563361	*D. melanogaster*	chrM:12877-12905:-		piRBase
DrMe_M_smithRNA128r	FR465521	*Drosophila*		7e-08	fRNAdb
DrMe_M_smithRNA128r	FR472905	*Drosophila*		4e-06	fRNAdb
DrMe_M_smithRNA128r	FR491761	*Drosophila*		2e-05	fRNAdb
DrMe_M_smithRNA128r	FR461400	*Drosophila*		2e-05	fRNAdb
DrMe_M_smithRNA128r	FR457376	*Drosophila*		2e-05	fRNAdb
DrMe_M_smithRNA128r	FR420437	*Drosophila*		2e-05	fRNAdb
DrMe_M_smithRNA128r	FR491760	*Drosophila*		7e-05	fRNAdb
DrMe_M_smithRNA128r	FR461399	*Drosophila*		7e-05	fRNAdb
DrMe_M_smithRNA128r	FR439103	*Drosophila*		7e-05	fRNAdb
DrMe_M_smithRNA128r	FR439102	*Drosophila*		7e-05	fRNAdb
DrMe_M_smithRNA128r	FR439101	*Drosophila*		7e-05	fRNAdb

DrMe_M_smithRNA128r	FR507766	*Drosophila*	3e-04	fRNAdb
DrMe_M_smithRNA128r	FR503730	*Drosophila*	3e-04	fRNAdb
DrMe_M_smithRNA128r	FR491759	*Drosophila*	3e-04	fRNAdb
DrMe_M_smithRNA128r	FR465520	*Drosophila*	3e-04	fRNAdb
DrMe_M_smithRNA128r	FR462906	*Drosophila*	3e-04	fRNAdb
DrMe_M_smithRNA128r	FR461398	*Drosophila*	3e-04	fRNAdb
DrMe_M_smithRNA128r	FR444019	*Drosophila*	3e-04	fRNAdb
DrMe_M_smithRNA128r	FR444018	*Drosophila*	3e-04	fRNAdb
DrMe_M_smithRNA128r	FR444017	*Drosophila*	3e-04	fRNAdb
DrMe_M_smithRNA128r	FR444016	*Drosophila*	3e-04	fRNAdb
DrMe_M_smithRNA128r	FR439100	*Drosophila*	3e-04	fRNAdb
DrMe_M_smithRNA128r	FR509191	*Drosophila*	0.001	fRNAdb
DrMe_M_smithRNA128r	FR507765	*Drosophila*	0.001	fRNAdb
DrMe_M_smithRNA128r	FR503729	*Drosophila*	0.001	fRNAdb
DrMe_M_smithRNA128r	FR492723	*Drosophila*	0.001	fRNAdb
DrMe_M_smithRNA128r	FR491758	*Drosophila*	0.001	fRNAdb
DrMe_M_smithRNA128r	FR466072	*Drosophila*	0.001	fRNAdb
DrMe_M_smithRNA128r	FR466071	*Drosophila*	0.001	fRNAdb
DrMe_M_smithRNA128r	FR465519	*Drosophila*	0.001	fRNAdb
DrMe_M_smithRNA128r	FR462905	*Drosophila*	0.001	fRNAdb
DrMe_M_smithRNA128r	FR462003	*Drosophila*	0.001	fRNAdb
DrMe_M_smithRNA128r	FR461397	*Drosophila*	0.001	fRNAdb
DrMe_M_smithRNA128r	FR444015	*Drosophila*	0.001	fRNAdb
DrMe_M_smithRNA128r	FR439099	*Drosophila*	0.001	fRNAdb
DrMe_M_smithRNA128r	FR509190	*Drosophila*	0.004	fRNAdb
DrMe_M_smithRNA128r	FR507764	*Drosophila*	0.004	fRNAdb
DrMe_M_smithRNA128r	FR503728	*Drosophila*	0.004	fRNAdb
DrMe_M_smithRNA128r	FR498186	*Drosophila*	0.004	fRNAdb
DrMe_M_smithRNA128r	FR492722	*Drosophila*	0.004	fRNAdb
DrMe_M_smithRNA128r	FR491757	*Drosophila*	0.004	fRNAdb
DrMe_M_smithRNA128r	FR472904	*Drosophila*	0.004	fRNAdb
DrMe_M_smithRNA128r	FR462904	*Drosophila*	0.004	fRNAdb
DrMe_M_smithRNA128r	FR461396	*Drosophila*	0.004	fRNAdb
DrMe_M_smithRNA128r	FR430554	*Drosophila*	0.004	fRNAdb
DrMe_M_smithRNA128r	FR420436	*Drosophila*	0.004	fRNAdb
DrMe_M_smithRNA128r	FR507763	*Drosophila*	0.016	fRNAdb
DrMe_M_smithRNA128r	FR503727	*Drosophila*	0.016	fRNAdb
DrMe_M_smithRNA128r	FR491756	*Drosophila*	0.016	fRNAdb
DrMe_M_smithRNA128r	FR462903	*Drosophila*	0.016	fRNAdb
DrMe_M_smithRNA128r	FR461395	*Drosophila*	0.016	fRNAdb
DrMe_M_smithRNA128r	FR420435	*Drosophila*	0.016	fRNAdb
DrMe_M_smithRNA128r	FR509189	*Drosophila*	0.065	fRNAdb

DrMe_M_smithRNA128r	FR507762	*Drosophila*	0.065	fRNAdb
DrMe_M_smithRNA128r	FR503726	*Drosophila*	0.065	fRNAdb
DrMe_M_smithRNA128r	FR462002	*Drosophila*	0.065	fRNAdb
DrMe_M_smithRNA128r	FR461394	*Drosophila*	0.065	fRNAdb
DrMe_M_smithRNA128r	FR509188	*Drosophila*	0.26	fRNAdb
DrMe_M_smithRNA128r	FR507761	*Drosophila*	0.26	fRNAdb
DrMe_M_smithRNA128r	FR503725	*Drosophila*	0.26	fRNAdb
DrMe_M_smithRNA128r	FR491755	*Drosophila*	0.26	fRNAdb
DrMe_M_smithRNA128r	FR461393	*Drosophila*	0.26	fRNAdb
DrMe_M_smithRNA128r	FR430553	*Drosophila*	0.26	fRNAdb
DrMe_M_smithRNA129rA	FR502574	*Drosophila*	0.002	fRNAdb
DrMe_M_smithRNA129rA	FR488828	*Drosophila*	0.002	fRNAdb
DrMe_M_smithRNA129rA	FR488827	*Drosophila*	0.002	fRNAdb
DrMe_M_smithRNA129rA	FR481546	*Drosophila*	0.002	fRNAdb
DrMe_M_smithRNA129rA	FR481545	*Drosophila*	0.002	fRNAdb
DrMe_M_smithRNA129rA	FR481544	*Drosophila*	0.002	fRNAdb
DrMe_M_smithRNA129rA	FR457625	*Drosophila*	0.002	fRNAdb
DrMe_M_smithRNA129rA	FR452260	*Drosophila*	0.002	fRNAdb
DrMe_M_smithRNA129rA	FR452259	*Drosophila*	0.002	fRNAdb
DrMe_M_smithRNA129rA	FR446084	*Drosophila*	0.002	fRNAdb
DrMe_M_smithRNA129rA	FR439370	*Drosophila*	0.002	fRNAdb
DrMe_M_smithRNA129rA	FR439369	*Drosophila*	0.002	fRNAdb
DrMe_M_smithRNA129rA	FR416755	*Drosophila*	0.002	fRNAdb
DrMe_M_smithRNA129rA	FR502573	*Drosophila*	0.006	fRNAdb
DrMe_M_smithRNA129rA	FR457624	*Drosophila*	0.006	fRNAdb
DrMe_M_smithRNA129rA	FR441277	*Drosophila*	0.006	fRNAdb
DrMe_M_smithRNA129rA	FR441276	*Drosophila*	0.006	fRNAdb
DrMe_M_smithRNA129rA	FR441275	*Drosophila*	0.006	fRNAdb
DrMe_M_smithRNA129rA	FR441274	*Drosophila*	0.006	fRNAdb
DrMe_M_smithRNA129rA	FR502572	*Drosophila*	0.025	fRNAdb
DrMe_M_smithRNA129rA	FR488826	*Drosophila*	0.025	fRNAdb
DrMe_M_smithRNA129rA	FR457623	*Drosophila*	0.025	fRNAdb
DrMe_M_smithRNA129rA	FR455331	*Drosophila*	0.025	fRNAdb
DrMe_M_smithRNA129rA	FR455069	*Drosophila*	0.025	fRNAdb
DrMe_M_smithRNA129rA	FR440428	*Drosophila*	0.025	fRNAdb
DrMe_M_smithRNA129rA	FR439368	*Drosophila*	0.025	fRNAdb
DrMe_M_smithRNA129rA	FR416754	*Drosophila*	0.025	fRNAdb
DrMe_M_smithRNA129rA	FR488825	*Drosophila*	0.099	fRNAdb
DrMe_M_smithRNA129rA	FR457622	*Drosophila*	0.099	fRNAdb
DrMe_M_smithRNA129rA	FR452258	*Drosophila*	0.099	fRNAdb
DrMe_M_smithRNA129rA	FR439367	*Drosophila*	0.099	fRNAdb
DrMe_M_smithRNA129rA	FR429489	*Drosophila*	0.099	fRNAdb

DrMe_M_smithRNA129rA	FR428291	*Drosophila*		0.099	fRNAdb
DrMe_M_smithRNA129rB	piR-dme-108224	*D. melanogaster*	chrM:12922-12945:-		piRBase
DrMe_M_smithRNA129rB	FR411819	*Drosophila*		1e-05	fRNAdb
DrMe_M_smithRNA129rB	FR411818	*Drosophila*		1e-05	fRNAdb
DrMe_M_smithRNA129rB	FR411817	*Drosophila*		1e-05	fRNAdb
DrMe_M_smithRNA129rB	FR411816	*Drosophila*		1e-05	fRNAdb
DrMe_M_smithRNA129rB	FR411815	*Drosophila*		1e-05	fRNAdb
DrMe_M_smithRNA129rB	FR409014	*Drosophila*		1e-05	fRNAdb
DrMe_M_smithRNA129rB	FR409013	*Drosophila*		1e-05	fRNAdb
DrMe_M_smithRNA129rB	FR409012	*Drosophila*		1e-05	fRNAdb
DrMe_M_smithRNA129rB	FR408118	*Drosophila*		1e-05	fRNAdb
DrMe_M_smithRNA129rB	FR408117	*Drosophila*		1e-05	fRNAdb
DrMe_M_smithRNA129rB	FR408116	*Drosophila*		1e-05	fRNAdb
DrMe_M_smithRNA129rB	FR407864	*Drosophila*		1e-05	fRNAdb
DrMe_M_smithRNA129rB	FR407863	*Drosophila*		1e-05	fRNAdb
DrMe_M_smithRNA129rB	FR407862	*Drosophila*		1e-05	fRNAdb
DrMe_M_smithRNA129rB	FR407776	*Drosophila*		1e-05	fRNAdb
DrMe_M_smithRNA129rB	FR423494	*Drosophila*		4e-05	fRNAdb
DrMe_M_smithRNA129rB	FR419887	*Drosophila*		4e-05	fRNAdb
DrMe_M_smithRNA129rB	FR419886	*Drosophila*		4e-05	fRNAdb
DrMe_M_smithRNA129rB	FR419885	*Drosophila*		4e-05	fRNAdb
DrMe_M_smithRNA129rB	FR419884	*Drosophila*		4e-05	fRNAdb
DrMe_M_smithRNA129rB	FR419883	*Drosophila*		4e-05	fRNAdb
DrMe_M_smithRNA129rB	FR411814	*Drosophila*		4e-05	fRNAdb
DrMe_M_smithRNA129rB	FR409011	*Drosophila*		4e-05	fRNAdb
DrMe_M_smithRNA129rB	FR408115	*Drosophila*		4e-05	fRNAdb
DrMe_M_smithRNA129rB	FR407775	*Drosophila*		4e-05	fRNAdb
DrMe_M_smithRNA129rB	FR455331	*Drosophila*		2e-04	fRNAdb
DrMe_M_smithRNA129rB	FR455330	*Drosophila*		2e-04	fRNAdb
DrMe_M_smithRNA129rB	FR455329	*Drosophila*		2e-04	fRNAdb
DrMe_M_smithRNA129rB	FR455328	*Drosophila*		2e-04	fRNAdb
DrMe_M_smithRNA129rB	FR455327	*Drosophila*		2e-04	fRNAdb
DrMe_M_smithRNA129rB	FR455326	*Drosophila*		2e-04	fRNAdb
DrMe_M_smithRNA129rB	FR423493	*Drosophila*		2e-04	fRNAdb
DrMe_M_smithRNA129rB	FR419882	*Drosophila*		2e-04	fRNAdb
DrMe_M_smithRNA129rB	FR411813	*Drosophila*		2e-04	fRNAdb
DrMe_M_smithRNA129rB	FR409010	*Drosophila*		2e-04	fRNAdb
DrMe_M_smithRNA129rB	FR408114	*Drosophila*		2e-04	fRNAdb
DrMe_M_smithRNA129rB	FR455325	*Drosophila*		6e-04	fRNAdb
DrMe_M_smithRNA129rB	FR429489	*Drosophila*		6e-04	fRNAdb
DrMe_M_smithRNA129rB	FR429488	*Drosophila*		6e-04	fRNAdb
DrMe_M_smithRNA129rB	FR429487	*Drosophila*		6e-04	fRNAdb

DrMe_M_smithRNA129rB	FR429486	*Drosophila*	6e-04	fRNAdb
DrMe_M_smithRNA129rB	FR429485	*Drosophila*	6e-04	fRNAdb
DrMe_M_smithRNA129rB	FR429484	*Drosophila*	6e-04	fRNAdb
DrMe_M_smithRNA129rB	FR419881	*Drosophila*	6e-04	fRNAdb
DrMe_M_smithRNA129rB	FR411812	*Drosophila*	6e-04	fRNAdb
DrMe_M_smithRNA129rB	FR409009	*Drosophila*	6e-04	fRNAdb
DrMe_M_smithRNA129rB	FR408113	*Drosophila*	6e-04	fRNAdb
DrMe_M_smithRNA129rB	FR502574	*Drosophila*	0.003	fRNAdb
DrMe_M_smithRNA129rB	FR502573	*Drosophila*	0.003	fRNAdb
DrMe_M_smithRNA129rB	FR502572	*Drosophila*	0.003	fRNAdb
DrMe_M_smithRNA129rB	FR502571	*Drosophila*	0.003	fRNAdb
DrMe_M_smithRNA129rB	FR502570	*Drosophila*	0.003	fRNAdb
DrMe_M_smithRNA129rB	FR502569	*Drosophila*	0.003	fRNAdb
DrMe_M_smithRNA129rB	FR502568	*Drosophila*	0.003	fRNAdb
DrMe_M_smithRNA129rB	FR502567	*Drosophila*	0.003	fRNAdb
DrMe_M_smithRNA129rB	FR455324	*Drosophila*	0.003	fRNAdb
DrMe_M_smithRNA129rB	FR453907	*Drosophila*	0.003	fRNAdb
DrMe_M_smithRNA129rB	FR429483	*Drosophila*	0.003	fRNAdb
DrMe_M_smithRNA129rB	FR419880	*Drosophila*	0.003	fRNAdb
DrMe_M_smithRNA129rB	FR411811	*Drosophila*	0.003	fRNAdb
DrMe_M_smithRNA129rB	FR409008	*Drosophila*	0.003	fRNAdb
DrMe_M_smithRNA129rB	FR408112	*Drosophila*	0.003	fRNAdb
DrMe_M_smithRNA129rB	FR407861	*Drosophila*	0.003	fRNAdb
DrMe_M_smithRNA129rB	FR407774	*Drosophila*	0.003	fRNAdb
DrMe_M_smithRNA129rB	FR502566	*Drosophila*	0.010	fRNAdb
DrMe_M_smithRNA129rB	FR488828	*Drosophila*	0.010	fRNAdb
DrMe_M_smithRNA129rB	FR488827	*Drosophila*	0.010	fRNAdb
DrMe_M_smithRNA129rB	FR488826	*Drosophila*	0.010	fRNAdb
DrMe_M_smithRNA129rB	FR488825	*Drosophila*	0.010	fRNAdb
DrMe_M_smithRNA129rB	FR488824	*Drosophila*	0.010	fRNAdb
DrMe_M_smithRNA129rB	FR488823	*Drosophila*	0.010	fRNAdb
DrMe_M_smithRNA129rB	FR488822	*Drosophila*	0.010	fRNAdb
DrMe_M_smithRNA129rB	FR488821	*Drosophila*	0.010	fRNAdb
DrMe_M_smithRNA129rB	FR488820	*Drosophila*	0.010	fRNAdb
DrMe_M_smithRNA129rB	FR455323	*Drosophila*	0.010	fRNAdb
DrMe_M_smithRNA129rB	FR429482	*Drosophila*	0.010	fRNAdb
DrMe_M_smithRNA129rB	FR419879	*Drosophila*	0.010	fRNAdb
DrMe_M_smithRNA129rB	FR411810	*Drosophila*	0.010	fRNAdb
DrMe_M_smithRNA129rB	FR409007	*Drosophila*	0.010	fRNAdb
DrMe_M_smithRNA129rB	FR408111	*Drosophila*	0.010	fRNAdb
DrMe_M_smithRNA129rB	FR407860	*Drosophila*	0.010	fRNAdb
DrMe_M_smithRNA129rB	FR502565	*Drosophila*	0.040	fRNAdb

DrMe_M_smithRNA129rB	FR457625	*Drosophila*		0.040	fRNAdb
DrMe_M_smithRNA129rB	FR457624	*Drosophila*		0.040	fRNAdb
DrMe_M_smithRNA129rB	FR457623	*Drosophila*		0.040	fRNAdb
DrMe_M_smithRNA129rB	FR457622	*Drosophila*		0.040	fRNAdb
DrMe_M_smithRNA129rB	FR457621	*Drosophila*		0.040	fRNAdb
DrMe_M_smithRNA129rB	FR457620	*Drosophila*		0.040	fRNAdb
DrMe_M_smithRNA129rB	FR457619	*Drosophila*		0.040	fRNAdb
DrMe_M_smithRNA129rB	FR457618	*Drosophila*		0.040	fRNAdb
DrMe_M_smithRNA129rB	FR453906	*Drosophila*		0.040	fRNAdb
DrMe_M_smithRNA129rB	FR441001	*Drosophila*		0.040	fRNAdb
DrMe_M_smithRNA129rB	FR425908	*Drosophila*		0.040	fRNAdb
DrMe_M_smithRNA129rB	FR419878	*Drosophila*		0.040	fRNAdb
DrMe_M_smithRNA129rB	FR409006	*Drosophila*		0.040	fRNAdb
DrMe_M_smithRNA129rB	FR407859	*Drosophila*		0.040	fRNAdb
DrMe_M_smithRNA129rB	FR440428	*Drosophila*		0.16	fRNAdb
DrMe_M_smithRNA129rB	FR440427	*Drosophila*		0.16	fRNAdb
DrMe_M_smithRNA129rB	FR440426	*Drosophila*		0.16	fRNAdb
DrMe_M_smithRNA129rB	FR440425	*Drosophila*		0.16	fRNAdb
DrMe_M_smithRNA129rB	FR408110	*Drosophila*		0.16	fRNAdb
DrMe_M_smithRNA129rB	FR407858	*Drosophila*		0.16	fRNAdb
DrMe_M_smithRNA129rB	piR-hsa-1847	*Human*	chrM:2971-2999:+	0.16	fRNAdb
DrMe_M_smithRNA129rB	piR-hsa-2155	*Human*	chrM:2966-2997:+	0.16	fRNAdb
DrMe_M_smithRNA132r	FR490193	*Drosophila*		0.005	fRNAdb
DrMe_M_smithRNA132r	FR490192	*Drosophila*		0.005	fRNAdb
DrMe_M_smithRNA132r	FR490191	*Drosophila*		0.005	fRNAdb
DrMe_M_smithRNA132r	FR490190	*Drosophila*		0.005	fRNAdb
DrMe_M_smithRNA132r	FR460825	*Drosophila*		0.005	fRNAdb
DrMe_M_smithRNA132r	FR460824	*Drosophila*		0.005	fRNAdb
DrMe_M_smithRNA132r	FR460823	*Drosophila*		0.005	fRNAdb
DrMe_M_smithRNA132r	FR460822	*Drosophila*		0.005	fRNAdb
DrMe_M_smithRNA132r	FR460821	*Drosophila*		0.005	fRNAdb
DrMe_M_smithRNA132r	FR459462	*Drosophila*		0.005	fRNAdb
DrMe_M_smithRNA132r	FR459461	*Drosophila*		0.005	fRNAdb
DrMe_M_smithRNA132r	FR459460	*Drosophila*		0.005	fRNAdb
DrMe_M_smithRNA132r	FR459459	*Drosophila*		0.005	fRNAdb
DrMe_M_smithRNA132r	FR459458	*Drosophila*		0.005	fRNAdb
DrMe_M_smithRNA132r	FR459457	*Drosophila*		0.005	fRNAdb
DrMe_M_smithRNA132r	FR459456	*Drosophila*		0.005	fRNAdb
DrMe_M_smithRNA132r	FR459455	*Drosophila*		0.005	fRNAdb
DrMe_M_smithRNA132r	FR458886	*Drosophila*		0.005	fRNAdb
DrMe_M_smithRNA132r	FR458885	*Drosophila*		0.005	fRNAdb
DrMe_M_smithRNA132r	FR458884	*Drosophila*		0.005	fRNAdb

DrMe_M_smithRNA132r	FR458883	*Drosophila*	0.005	fRNAdb
DrMe_M_smithRNA132r	FR458882	*Drosophila*	0.005	fRNAdb
DrMe_M_smithRNA132r	FR451370	*Drosophila*	0.005	fRNAdb
DrMe_M_smithRNA132r	FR451369	*Drosophila*	0.005	fRNAdb
DrMe_M_smithRNA132r	FR447729	*Drosophila*	0.005	fRNAdb
DrMe_M_smithRNA132r	FR447728	*Drosophila*	0.005	fRNAdb
DrMe_M_smithRNA132r	FR447727	*Drosophila*	0.005	fRNAdb
DrMe_M_smithRNA132r	FR421706	*Drosophila*	0.005	fRNAdb
DrMe_M_smithRNA132r	FR421705	*Drosophila*	0.005	fRNAdb
DrMe_M_smithRNA132r	FR421704	*Drosophila*	0.005	fRNAdb
DrMe_M_smithRNA132r	FR421703	*Drosophila*	0.005	fRNAdb
DrMe_M_smithRNA132r	FR412445	*Drosophila*	0.005	fRNAdb
DrMe_M_smithRNA132r	FR412444	*Drosophila*	0.005	fRNAdb
DrMe_M_smithRNA132r	FR412443	*Drosophila*	0.005	fRNAdb
DrMe_M_smithRNA132r	FR501375	*Drosophila*	0.020	fRNAdb
DrMe_M_smithRNA132r	FR460820	*Drosophila*	0.020	fRNAdb
DrMe_M_smithRNA132r	FR451662	*Drosophila*	0.020	fRNAdb
DrMe_M_smithRNA132r	FR451661	*Drosophila*	0.020	fRNAdb
DrMe_M_smithRNA132r	FR421702	*Drosophila*	0.020	fRNAdb
DrMe_M_smithRNA132r	FR460819	*Drosophila*	0.079	fRNAdb
DrMe_M_smithRNA132r	FR458881	*Drosophila*	0.079	fRNAdb
DrMe_M_smithRNA132r	FR451368	*Drosophila*	0.079	fRNAdb
DrMe_M_smithRNA132r	FR447726	*Drosophila*	0.079	fRNAdb
DrMe_M_smithRNA132r	FR421701	*Drosophila*	0.079	fRNAdb
DrMe_M_smithRNA132r	FR414770	*Drosophila*	0.079	fRNAdb
DrMe_M_smithRNA132r	FR414006	*Drosophila*	0.079	fRNAdb
DrMe_M_smithRNA132r	FR414005	*Drosophila*	0.079	fRNAdb
DrMe_M_smithRNA132r	FR414004	*Drosophila*	0.079	fRNAdb
DrMe_M_smithRNA132r	FR414003	*Drosophila*	0.079	fRNAdb
DrMe_M_smithRNA132r	FR414002	*Drosophila*	0.079	fRNAdb
DrMe_M_smithRNA132r	FR412442	*Drosophila*	0.079	fRNAdb
DrMe_M_smithRNA132r	FR460818	*Drosophila*	0.31	fRNAdb
DrMe_M_smithRNA132r	FR458880	*Drosophila*	0.31	fRNAdb
DrMe_M_smithRNA132r	FR426664	*Drosophila*	0.31	fRNAdb
DrMe_M_smithRNA132r	FR426663	*Drosophila*	0.31	fRNAdb
DrMe_M_smithRNA132r	FR426662	*Drosophila*	0.31	fRNAdb
DrMe_M_smithRNA132r	FR426661	*Drosophila*	0.31	fRNAdb
DrMe_M_smithRNA132r	FR426660	*Drosophila*	0.31	fRNAdb
DrMe_M_smithRNA132r	FR426659	*Drosophila*	0.31	fRNAdb
DrMe_M_smithRNA132r	FR421700	*Drosophila*	0.31	fRNAdb
DrMe_M_smithRNA132r	FR412441	*Drosophila*	0.31	fRNAdb
DrMe_M_smithRNA133rA	FR441631	*Drosophila*	4e-05	fRNAdb

DrMe_M_smithRNA133rA	FR463301	*Drosophila*	1e-04	fRNAdb
DrMe_M_smithRNA133rA	FR463300	*Drosophila*	1e-04	fRNAdb
DrMe_M_smithRNA133rA	FR463299	*Drosophila*	1e-04	fRNAdb
DrMe_M_smithRNA133rA	FR463298	*Drosophila*	1e-04	fRNAdb
DrMe_M_smithRNA133rA	FR463297	*Drosophila*	1e-04	fRNAdb
DrMe_M_smithRNA133rA	FR463296	*Drosophila*	1e-04	fRNAdb
DrMe_M_smithRNA133rA	FR422480	*Drosophila*	1e-04	fRNAdb
DrMe_M_smithRNA133rA	FR476803	*Drosophila*	6e-04	fRNAdb
DrMe_M_smithRNA133rA	FR476802	*Drosophila*	6e-04	fRNAdb
DrMe_M_smithRNA133rA	FR476801	*Drosophila*	6e-04	fRNAdb
DrMe_M_smithRNA133rA	FR463295	*Drosophila*	6e-04	fRNAdb
DrMe_M_smithRNA133rA	FR422479	*Drosophila*	6e-04	fRNAdb
DrMe_M_smithRNA133rA	FR490968	*Drosophila*	0.002	fRNAdb
DrMe_M_smithRNA133rA	FR476800	*Drosophila*	0.002	fRNAdb
DrMe_M_smithRNA133rA	FR463294	*Drosophila*	0.002	fRNAdb
DrMe_M_smithRNA133rA	FR460466	*Drosophila*	0.002	fRNAdb
DrMe_M_smithRNA133rA	FR430519	*Drosophila*	0.002	fRNAdb
DrMe_M_smithRNA133rA	FR430518	*Drosophila*	0.002	fRNAdb
DrMe_M_smithRNA133rA	FR430517	*Drosophila*	0.002	fRNAdb
DrMe_M_smithRNA133rA	FR430516	*Drosophila*	0.002	fRNAdb
DrMe_M_smithRNA133rA	FR430515	*Drosophila*	0.002	fRNAdb
DrMe_M_smithRNA133rA	FR430514	*Drosophila*	0.002	fRNAdb
DrMe_M_smithRNA133rA	FR430513	*Drosophila*	0.002	fRNAdb
DrMe_M_smithRNA133rA	FR430512	*Drosophila*	0.002	fRNAdb
DrMe_M_smithRNA133rA	FR427608	*Drosophila*	0.002	fRNAdb
DrMe_M_smithRNA133rA	FR422478	*Drosophila*	0.002	fRNAdb
DrMe_M_smithRNA133rA	FR508945	*Drosophila*	0.009	fRNAdb
DrMe_M_smithRNA133rA	FR508944	*Drosophila*	0.009	fRNAdb
DrMe_M_smithRNA133rA	FR508943	*Drosophila*	0.009	fRNAdb
DrMe_M_smithRNA133rA	FR508942	*Drosophila*	0.009	fRNAdb
DrMe_M_smithRNA133rA	FR508941	*Drosophila*	0.009	fRNAdb
DrMe_M_smithRNA133rA	FR463293	*Drosophila*	0.009	fRNAdb
DrMe_M_smithRNA133rA	FR460465	*Drosophila*	0.009	fRNAdb
DrMe_M_smithRNA133rA	FR430511	*Drosophila*	0.009	fRNAdb
DrMe_M_smithRNA133rA	FR506935	*Drosophila*	0.035	fRNAdb
DrMe_M_smithRNA133rA	FR506934	*Drosophila*	0.035	fRNAdb
DrMe_M_smithRNA133rA	FR506933	*Drosophila*	0.035	fRNAdb
DrMe_M_smithRNA133rA	FR506932	*Drosophila*	0.035	fRNAdb
DrMe_M_smithRNA133rA	FR506931	*Drosophila*	0.035	fRNAdb
DrMe_M_smithRNA133rA	FR490967	*Drosophila*	0.035	fRNAdb
DrMe_M_smithRNA133rA	FR460464	*Drosophila*	0.035	fRNAdb
DrMe_M_smithRNA133rA	FR441630	*Drosophila*	0.035	fRNAdb

DrMe_M_smithRNA133rA	FR438629	*Drosophila*		0.035	fRNAdb
DrMe_M_smithRNA133rA	FR427607	*Drosophila*		0.035	fRNAdb
DrMe_M_smithRNA133rA	FR414324	*Drosophila*		0.035	fRNAdb
DrMe_M_smithRNA133rA	FR501575	*Drosophila*		0.14	fRNAdb
DrMe_M_smithRNA133rA	FR501574	*Drosophila*		0.14	fRNAdb
DrMe_M_smithRNA133rA	FR501573	*Drosophila*		0.14	fRNAdb
DrMe_M_smithRNA133rA	FR501572	*Drosophila*		0.14	fRNAdb
DrMe_M_smithRNA133rA	FR490966	*Drosophila*		0.14	fRNAdb
DrMe_M_smithRNA133rA	FR441629	*Drosophila*		0.14	fRNAdb
DrMe_M_smithRNA133rA	FR438628	*Drosophila*		0.14	fRNAdb
DrMe_M_smithRNA133rB	piR-dme-1094845	*D. melanogaster*	chrM:13370-13396:-		piRBase
DrMe_M_smithRNA133rB	FR481783	*Drosophila*		4e-06	fRNAdb
DrMe_M_smithRNA133rB	FR499875	*Drosophila*		1e-05	fRNAdb
DrMe_M_smithRNA133rB	FR441956	*Drosophila*		1e-05	fRNAdb
DrMe_M_smithRNA133rB	FR428980	*Drosophila*		1e-05	fRNAdb
DrMe_M_smithRNA133rB	FR499874	*Drosophila*		6e-05	fRNAdb
DrMe_M_smithRNA133rB	FR481782	*Drosophila*		6e-05	fRNAdb
DrMe_M_smithRNA133rB	FR476137	*Drosophila*		6e-05	fRNAdb
DrMe_M_smithRNA133rB	FR428979	*Drosophila*		6e-05	fRNAdb
DrMe_M_smithRNA133rB	FR499873	*Drosophila*		2e-04	fRNAdb
DrMe_M_smithRNA133rB	FR476136	*Drosophila*		2e-04	fRNAdb
DrMe_M_smithRNA133rB	FR441955	*Drosophila*		2e-04	fRNAdb
DrMe_M_smithRNA133rB	FR428978	*Drosophila*		2e-04	fRNAdb
DrMe_M_smithRNA133rB	FR481781	*Drosophila*		9e-04	fRNAdb
DrMe_M_smithRNA133rB	FR476135	*Drosophila*		9e-04	fRNAdb
DrMe_M_smithRNA133rB	FR428977	*Drosophila*		9e-04	fRNAdb
DrMe_M_smithRNA133rB	FR476134	*Drosophila*		0.004	fRNAdb
DrMe_M_smithRNA133rB	FR448500	*Drosophila*		0.004	fRNAdb
DrMe_M_smithRNA133rB	FR441954	*Drosophila*		0.004	fRNAdb
DrMe_M_smithRNA133rB	FR428976	*Drosophila*		0.004	fRNAdb
DrMe_M_smithRNA133rB	FR424963	*Drosophila*		0.004	fRNAdb
DrMe_M_smithRNA133rB	FR476133	*Drosophila*		0.014	fRNAdb
DrMe_M_smithRNA133rB	FR469959	*Drosophila*		0.014	fRNAdb
DrMe_M_smithRNA133rB	FR441953	*Drosophila*		0.014	fRNAdb
DrMe_M_smithRNA133rB	FR428975	*Drosophila*		0.014	fRNAdb
DrMe_M_smithRNA133rB	FR423893	*Drosophila*		0.014	fRNAdb
DrMe_M_smithRNA133rB	FR413549	*Drosophila*		0.014	fRNAdb
DrMe_M_smithRNA133rB	FR476132	*Drosophila*		0.055	fRNAdb
DrMe_M_smithRNA133rB	FR469958	*Drosophila*		0.055	fRNAdb
DrMe_M_smithRNA133rB	FR424962	*Drosophila*		0.055	fRNAdb
DrMe_M_smithRNA133rB	FR423892	*Drosophila*		0.055	fRNAdb
DrMe_M_smithRNA133rB	FR413548	*Drosophila*		0.055	fRNAdb

DrMe_M_smithRNA133rB	FR469957	*Drosophila*		0.22	fRNAdb
DrMe_M_smithRNA133rB	FR424961	*Drosophila*		0.22	fRNAdb
DrMe_M_smithRNA133rB	FR423891	*Drosophila*		0.22	fRNAdb
DrMe_M_smithRNA133rB	FR413547	*Drosophila*		0.22	fRNAdb
DrMe_M_smithRNA133rB	FR413216	*Drosophila*		0.22	fRNAdb
DrMe_M_smithRNA133rB	FR409498	*Drosophila*		0.22	fRNAdb
DrMe_M_smithRNA133rB	FR408261	*Drosophila*		0.22	fRNAdb
DrMe_M_smithRNA141r	piR-dme-1955501	*D. melanogaster*	chrM:14176-14205:-		piRBase
DrMe_M_smithRNA141r	FR439019	*Drosophila*		2e-05	fRNAdb
DrMe_M_smithRNA141r	FR481729	*Drosophila*		7e-05	fRNAdb
DrMe_M_smithRNA141r	FR443572	*Drosophila*		7e-05	fRNAdb
DrMe_M_smithRNA141r	FR481898	*Drosophila*		3e-04	fRNAdb
DrMe_M_smithRNA141r	FR481728	*Drosophila*		3e-04	fRNAdb
DrMe_M_smithRNA141r	FR463902	*Drosophila*		3e-04	fRNAdb
DrMe_M_smithRNA141r	FR443571	*Drosophila*		3e-04	fRNAdb
DrMe_M_smithRNA141r	FR442257	*Drosophila*		3e-04	fRNAdb
DrMe_M_smithRNA141r	FR481897	*Drosophila*		0.001	fRNAdb
DrMe_M_smithRNA141r	FR463901	*Drosophila*		0.001	fRNAdb
DrMe_M_smithRNA141r	FR441808	*Drosophila*		0.001	fRNAdb
DrMe_M_smithRNA141r	FR435815	*Drosophila*		0.001	fRNAdb
DrMe_M_smithRNA141r	FR481896	*Drosophila*		0.004	fRNAdb
DrMe_M_smithRNA141r	FR481727	*Drosophila*		0.004	fRNAdb
DrMe_M_smithRNA141r	FR446541	*Drosophila*		0.004	fRNAdb
DrMe_M_smithRNA141r	FR442256	*Drosophila*		0.004	fRNAdb
DrMe_M_smithRNA141r	FR439018	*Drosophila*		0.004	fRNAdb
DrMe_M_smithRNA141r	FR435814	*Drosophila*		0.004	fRNAdb
DrMe_M_smithRNA141r	FR426006	*Drosophila*		0.004	fRNAdb
DrMe_M_smithRNA141r	FR442255	*Drosophila*		0.018	fRNAdb
DrMe_M_smithRNA141r	FR435813	*Drosophila*		0.018	fRNAdb
DrMe_M_smithRNA141r	FR416077	*Drosophila*		0.018	fRNAdb
DrMe_M_smithRNA141r	FR448784	*Drosophila*		0.070	fRNAdb
DrMe_M_smithRNA141r	FR448783	*Drosophila*		0.070	fRNAdb
DrMe_M_smithRNA141r	FR448782	*Drosophila*		0.070	fRNAdb
DrMe_M_smithRNA141r	FR435812	*Drosophila*		0.070	fRNAdb
DrMe_M_smithRNA141r	FR416076	*Drosophila*		0.070	fRNAdb
DrMe_M_smithRNA141r	FR462625	*Drosophila*		0.27	fRNAdb
DrMe_M_smithRNA141r	FR435811	*Drosophila*		0.27	fRNAdb
DrMe_M_smithRNA141r	FR432800	*Drosophila*		0.27	fRNAdb
DrMe_M_smithRNA141r	FR416075	*Drosophila*		0.27	fRNAdb
DrMe_M_smithRNA141r	FR415496	*Drosophila*		0.27	fRNAdb
DrMe_M_smithRNA144r	piR-dme-671956	*D. melanogaster*	chrM:14396-14420:-		piRBase
DrMe_M_smithRNA144r	FR471252	*Drosophila*		5e-05	fRNAdb

DrMe_M_smithRNA144r	FR424101	*Drosophila*		2e-04	fRNAdb
DrMe_M_smithRNA144r	FR443602	*Drosophila*		7e-04	fRNAdb
DrMe_M_smithRNA144r	FR443601	*Drosophila*		7e-04	fRNAdb
DrMe_M_smithRNA144r	FR423402	*Drosophila*		7e-04	fRNAdb
DrMe_M_smithRNA144r	FR464001	*Drosophila*		0.003	fRNAdb
DrMe_M_smithRNA144r	FR443600	*Drosophila*		0.003	fRNAdb
DrMe_M_smithRNA144r	FR424100	*Drosophila*		0.003	fRNAdb
DrMe_M_smithRNA144r	FR416696	*Drosophila*		0.003	fRNAdb
DrMe_M_smithRNA144r	FR505546	*Drosophila*		0.011	fRNAdb
DrMe_M_smithRNA144r	FR497451	*Drosophila*		0.011	fRNAdb
DrMe_M_smithRNA144r	FR478656	*Drosophila*		0.011	fRNAdb
DrMe_M_smithRNA144r	FR424099	*Drosophila*		0.011	fRNAdb
DrMe_M_smithRNA144r	FR423401	*Drosophila*		0.011	fRNAdb
DrMe_M_smithRNA144r	FR505545	*Drosophila*		0.045	fRNAdb
DrMe_M_smithRNA144r	FR497450	*Drosophila*		0.045	fRNAdb
DrMe_M_smithRNA144r	FR474437	*Drosophila*		0.045	fRNAdb
DrMe_M_smithRNA144r	FR467085	*Drosophila*		0.045	fRNAdb
DrMe_M_smithRNA144r	FR424666	*Drosophila*		0.045	fRNAdb
DrMe_M_smithRNA144r	FR423400	*Drosophila*		0.045	fRNAdb
DrMe_M_smithRNA144r	FR497449	*Drosophila*		0.18	fRNAdb
DrMe_M_smithRNA144r	FR467084	*Drosophila*		0.18	fRNAdb
DrMe_M_smithRNA144r	FR425386	*Drosophila*		0.18	fRNAdb
MuMu_F_smithRNA11r	piR-mmu-55370144	*Mus musculus*	chrM:1093-1118:+		piRBase
MuMu_F_smithRNA156nc	piR-mmu-4681867	*Mus musculus*	chrM:15615-15642:-		piRBase
MuMu_F_smithRNA24r	piR-mmu-51313768	*Mus musculus*			piRBase
MuMu_F_smithRNA37t	piR-mmu-10841588	*Mus musculus*			piRBase
MuMu_F_smithRNA7r	piR-mmu-31083152	*Mus musculus*	chrM:779-809:+		piRBase
MuMu_M_smithRNA8r	piR-mmu-1076637	*Mus musculus*	chrM:838-866:+		piRBase

[1] Name of retrieved piRNA/miRNA, or fRNAdb Accession.

[2] When available.

Supplementary Table S5. GenBank Accession Numbers of complete mitochondrial genomes of *Drosophila melanogaster* and *Mus musculus*.

Species	GenBank Accession Number
Drosophila melanogaster	AF200828
	CM008274
	CM010505
	CM010512
	CM010519
	CM010526
	CM010533
	CM010540
	CM010547
	CM010554
	CM010561
	CM010568
	CM010575
	CM010582
	CM010589
	CM010596
	DMU37541
	JQ686693
	JQ686694
	KJ947872
	KP161877
	KP843842
	KP843843
	KP843844
	KP843845
	KP843846
	KP843847
	KP843848
	KP843849
	KP843850
	KP843851
	KP843852
	KP843853
	KP843854
	KT174472
	KT174473
	KT174474
	KT896664

	KU764535
	KY310613
	KY310614
	KY310615
	NC_024511
Mus musculus	AY172335
	AY339599
	AY466499
	AY533105
	AY533106
	AY533107
	AY533108
	AY675564
	AY999076
	CM004178
	CM004179
	CM004180
	CM004181
	CM004182
	CM004183
	CM004184
	CM004185
	CM004186
	CM004187
	CM004189
	CM004214
	CM004255
	CM004276
	CM004277
	D83491
	DQ106412
	DQ106413
	DQ874614
	EF108330
	EF108331
	EF108332
	EF108333
	EF108334
	EF108335
	EF108336
	EF108337
	EF108338

EF108339
EF108340
EF108341
EF108342
EF108343
EF108344
EF108345
EU312160
EU312161
EU315228
EU315229
EU450583
FJ374639
FJ374640
FJ374641
FJ374642
FJ374643
FJ374644
FJ374645
FJ374646
FJ374647
FJ374648
FJ374649
FJ374650
FJ374651
FJ374652
FJ374653
FJ374654
FJ374655
FJ374656
FJ374657
FJ374658
FJ374659
FJ374660
FJ374661
FJ374662
FJ374663
FJ374664
FJ374665
FJ803909
GQ871744
GQ871745

GQ871746
HQ586004
HQ675026
HQ675027
HQ675028
HQ675029
HQ675030
HQ675031
HQ877407
J01420
JF286601
JQ003190
JX945964
JX945965
JX945966
JX945967
JX945968
JX945969
JX945970
JX945971
JX945972
JX945973
JX945974
JX945975
JX945976
JX945977
JX945978
JX945979
KC663618
KC663619
KC663620
KC663621
KC663622
KF781645
KF781646
KF781647
KF781648
KF781649
KF781650
KF781651
KF781652
KF781653

KF781654
KF781655
KF781656
KF781657
KF781658
KF781659
KF781660
KF781661
KF781662
KF781663
KF781664
KF937873
KF937874
KF937875
KF937876
KP090294
KP260514
KP260515
KP260516
KP260517
KP877610
KP877611
KP877612
KP877613
KP877614
KP877615
KP877616
KP877617
KP877618
KP877619
KP877620
KR020497
KR020498
KR020499
KR020500
KY018919
NC_005089
NC_006914
NC_006915
NC_010339
NC_012387

Supplementary Table S6. PCR primers.

Primer name	Sequence	Reference
MLUR_3604	5’-AGTTTTCAAAGCTCTATATTTTGGGTTA-3’	[50]
MLUR_4442	5’-TTTTAAAACTTCATGGATTTCATTACAA-3’	[50]
RuPhM_p1_156F	5’-GCTCCTACTAGTAATATTTCTTTT-3’	This study
RuPhM_p1_1543R	5’-TTTTATGATTAAACTACCAAAACC-3’	This study
RuPhM_p3_12612F	5’-TTATGTTAAACGCAGATATAAGTA-3’	This study
RuPhM_p3_13106R	5’-AAAGAAAATTATTACCAATCCATG-3’	This study
RuPhM_p4_14438F	5’-GATAGGATTTGAAGATAAAACGTT-3’	This study
RuPhM_p4_14943R	5’-AAAATAACAATAACCCTAACCTAA-3’	This study
RuPhF_p1_169F	5’-GTCTTCCTATTTAATTTAGTGTTG-3’	This study
RuPhF_p1_1526R	5’-TTCTTTATTAAACTACCAAAACAC-3’	This study
RuPhF_p3_1285F	5’-TGAAATGAGGTCTTCTAATATAAG-3’	This study
RuPhF_p3_1921R	5’-CTTTAAAACCTAAGCCATTAAATA-3’	This study

Supplementary Table S7. SRA Accession Numbers of raw reads used in this study.

Dataset	SRA Accession Number
Ovary Replicate 1 sRNAseq; *Danio rerio*; miRNA-Seq	SRR1265755
Ovary Replicate 1 sRNAseq; *Danio rerio*; miRNA-Seq	SRR1265756
Ovary Replicate 1 sRNAseq; *Danio rerio*; miRNA-Seq	SRR1265757
Testis Replicate 1 sRNAseq; *Danio rerio*; miRNA-Seq	SRR1265758
Testis Replicate 1 sRNAseq; *Danio rerio*; miRNA-Seq	SRR1265759
Testis Replicate 1 sRNAseq; *Danio rerio*; miRNA-Seq	SRR1265760
ovary_rep1; *Danio rerio*; RNA-Seq	SRR6841457
ovary_rep1; *Danio rerio*; RNA-Seq	SRR6841458
ovary_rep1; *Danio rerio*; RNA-Seq	SRR6841459
ovary_rep2; *Danio rerio*; RNA-Seq	SRR6841460
ovary_rep2; *Danio rerio*; RNA-Seq	SRR6841461
ovary_rep2; *Danio rerio*; RNA-Seq	SRR6841462
testis_rep1; *Danio rerio*; RNA-Seq	SRR6841469
testis_rep1; *Danio rerio*; RNA-Seq	SRR6841470
testis_rep1; *Danio rerio*; RNA-Seq	SRR6841471
testis_rep2; *Danio rerio*; RNA-Seq	SRR6841472
testis_rep2; *Danio rerio*; RNA-Seq	SRR6841473
testis_rep2; *Danio rerio*; RNA-Seq	SRR6841474
smallRNA X^X (control) ovary Replica 1; *Drosophila melanogaster*; ncRNA-Seq	SRR5181429
smallRNA X^X (control) ovary Replica 2; *Drosophila melanogaster*; ncRNA-Seq	SRR5181430
smallRNA X^Y (control) testis Replica 1; *Drosophila melanogaster*; ncRNA-Seq	SRR5181435
smallRNA X^Y (control) testis Replica 2; *Drosophila melanogaster*; ncRNA-Seq	SRR5181436
mRNA X^X (control) ovary Replica 1; *Drosophila melanogaster*; RNA-Seq	SRR5181417
mRNA X^X (control) ovary Replica 2; *Drosophila melanogaster*; RNA-Seq	SRR5181418
mRNA X^Y (control) testis Replica 1; *Drosophila melanogaster*; RNA-Seq	SRR5181423
mRNA X^Y (control) testis Replica 2; *Drosophila melanogaster*; RNA-Seq	SRR5181424
Illumina HiSeq 1500 sequencing of SAMD00048521	DRR083999
Illumina HiSeq 1500 sequencing of SAMD00048521	DRR084000
Illumina HiSeq 1500 sequencing of SAMD00048521	DRR084001
Illumina HiSeq 1500 sequencing of SAMD00048521	DRR084005
Illumina HiSeq 1500 sequencing of SAMD00048521	DRR084006
Illumina HiSeq 1500 sequencing of SAMD00048521	DRR084007
Illumina HiSeq 1500 paired end sequencing of SAMD00048521	DRR084021
Illumina HiSeq 1500 paired end sequencing of SAMD00048521	DRR084022
Illumina HiSeq 1500 paired end sequencing of SAMD00048521	DRR084024
Illumina HiSeq 1500 paired end sequencing of SAMD00048521	DRR084031
Illumina HiSeq 1500 sequencing of SAMD00048521	DRR084032
Illumina HiSeq 1500 sequencing of SAMD00048521	DRR084033
P14_Con_s1; *Mus musculus*; ncRNA-Seq	SRR3521024

P14_Con_s2; *Mus musculus*; ncRNA-Seq	SRR3521025
P14_Con_1; *Mus musculus*; RNA-Seq	SRR3520994
P14_Con_2; *Mus musculus*; RNA-Seq	SRR3520995
